# Maternal diet associated with infants’ intestinal microbiota mediated by predominant long-chain fatty acid in breast milk

**DOI:** 10.3389/fmicb.2022.1004175

**Published:** 2023-01-06

**Authors:** Menglu Xi, Xiaona Na, Xia Ma, Hanglian Lan, Ting Sun, Wei-Hsien Liu, Weilian Hung, Ai Zhao

**Affiliations:** ^1^Vanke School of Public Health, Tsinghua University, Beijing, China; ^2^Inner Mongolia Dairy Technology Research Institute Co., Ltd., Hohhot, China; ^3^Yili Innovation Center, Inner Mongolia Yili Industrial Group Co., Ltd., Hohhot, China

**Keywords:** long-chain fatty acids, breast milk, metagenomics sequencing, infant microbiota, KEGG

## Abstract

**Introduction:**

Long-chain fatty acids in breast milk are affected by the mother’s diet and play an important role in the growth, development, and immune construction of infants. This study aims to explore the correlation between maternal diet, breast milk fatty acids (FAs), and the infant intestinal flora.

**Methods:**

We enrolled 56 paired mothers and their infants; both breast milk samples and infants’ fecal samples were collected to determine the long-chain FA content of breast milk by ultra-performance liquid chromatography–tandem mass spectrometry (UPLC-MS), and metagenomic technology was applied to determine the microbial composition of infant feces. The maternal diet was also investigated using a 24-h dietary recall.

**Results:**

The results indicated that the fat contribution rates of edible oils in the maternal diet are significantly positively correlated with the contents of certain long-chain fatty acids (C16:0, C18:1, C16:1, and C22:4) in breast milk, which mainly regulate the abundance of *Lacticaseibacillus rhamnosus, Lacticaseibacillus fermentum*, and *Lacticaseibacillus paracasei* in the infant gut. Through KEGG pathway analysis, our data revealed that the long-chain FAs in different groups of breast milk were significantly correlated with the pathways of biotin metabolism, glycerolipid metabolism, and starch and sucrose metabolism.

**Discussion:**

The results of this study suggest a pathway in which the diets of lactating mothers may affect the composition of the infant intestinal microbiota by influencing breast milk FAs and then further regulating infant health.

## Introduction

Human breast milk is considered optimal nutrition for infants, providing essential nutrients and a broad range of bioactive compounds, as well as supporting colonization by the gut microbiota, which is conducive to infant growth and development (Li et al., 2019; Thai and Gregory, 2020; Yan et al., 2021).

Breast milk contains 5% fat, which is the main energy-yielding nutrient. The most abundant type of fat in breast milk is triglycerides, which contain one glycerol linked to three fatty acid (FA) moieties. Long-chain FAs refer to FAs with 14 to 26 carbon atoms in their structure and account for more than 90% of the FA content in breast milk. It is well-documented that long-chain FAs in human milk play a crucial role in infant growth and development (Bobiński and Bobińska, 2020). For example, oleic acid (18:1), one of the long-chain FAs, can provide the main energy supply for newborns to ensure the rapid growth and development of the body’s tissues and organs (Sánchez-Hernández et al., 2019). Accordingly, it accounts for the highest proportion of FAs human milk, ranging from 38 to 40% (Sánchez-Hernández et al., 2019; Bobiński and Bobińska, 2020). In addition, many long-chain FAs also have certain biological functions; for example, docosahexaenoic acid (DHA, C22:6) and eicosatetraenoic acid (ARA, C20:4) benefit the health of the infant brain, eyes, and heart (Mun et al., 2019). Therefore, obtaining a rich supply of various fatty acids in early life is critical for infant health and development, and breast milk is the sole source of these FAs for exclusively breastfed babies. The content of long-chain FAs in breast milk relies highly on maternal nutritional status and food intake (Delplanque et al., 2015). Population-based studies have demonstrated that varied dietary fats, such as docosahexaenoic acid (DHA) and other polyunsaturated fatty acids (PUFA) and monounsaturated fatty acids (MUFA), all contribute to the long-chain FA composition of breast milk (Finley et al., 1985; Antonakou et al., 2013; Daud et al., 2013; Mäkelä et al., 2013).

With the development of Illumina sequencing, research shows that in addition to nutrients, highly diversified bacterial taxa are present in human milk (Li et al., 2017). Human milk is thus considered the main source used by newborns to establish a healthy microbiome (Andreas et al., 2015; Toscano et al., 2017; Zimmermann and Curtis, 2020). It has been documented that breast milk not only contains flora but is also a complete biological system: certain long-chain FAs in breast milk also play an important regulatory role in the colonization of the infant intestinal flora (Pannaraj et al., 2017; Nyangahu and Jaspan, 2019). Niers et al. (2007) found that palmitic acid (C16:0) in breast milk can affect the composition of the infant intestinal flora and promote the development of infant intestinal function. Jiang et al. found that C14:0, C18:0, C16:0, C20:4, and C22:6 were correlated with *Bacteroides, Veillonella, Streptococcus*, and *Clostridium* (Jiang et al., 2018). The associations between long-chain FAs and infants’ microbiota were also confirmed by interventional study. Yaron et al. (2013) investigated infants fed infant formula rich in C16:0 and reported that the number of *Lactobacillus* and *Bifidobacteria* in infant feces increased after 6 weeks. The above studies show that certain long-chain FAs may act as prebiotics, which benefit the colonization of the infant gut microbiota. However, the studies were very limited, and most previous studies focused on specific FAs and specific microbes. It is of great significance to elucidate the whole picture of which long-chain FAs in breast milk could be targeted to regulate the levels of microorganisms in the infant gut. Moreover, as mentioned above, since diet is considered as an important factor affecting the composition of long-chain FAs in breast milk, it is interesting to know whether the maternal diet could influence the infant’s gut health *via* breast milk.

This study aimed to explore the pathway from maternal dietary intake to long-chain FAs composition in breast milk and the infant intestinal microbiota, to provide scientific guidance for a rational diet for lactating mothers and a theoretical reference for healthier infant growth.

## Materials and methods

### Ethical concerns

All participants gave written informed consent following the Declaration of Helsinki. The protocol was approved by the Research Center for Public Health, Tsinghua University (NO.THUSM/PHREC/2021–003).

### Study design

We recruited 56 paired mothers and their infants from three cities in China (Xuchang, Cangzhou, and Chenzhou, *N* = 20, 18, and 18, respectively). The inclusion criteria for infants were (1) singleton, (2) full-term (≥37 weeks), (3) 30–120 days old, (4) exclusive breastfeeding, (5) birth weight range from 2.5 to 4 kg, (6) without disability or any diagnosed disease at birth, and (7) Apgar score ≥ 8. Infants who had used any prebiotics, probiotics, and antibiotics in the past 4 weeks were excluded. Mothers in the age range from 20 to 45 years were included and were excluded if they had suffered from mastitis in the past 4 weeks or had used any prebiotics, probiotics, or antibiotics in the past 4 weeks.

### Sample collection

#### Data collection

An interviewer-administered questionnaire was used to collect data on socio-demographic characteristics, lifestyle and behavioral information, history of pregnancy and delivery, and dietary intake. Short-term dietary intake assessment was based on a one-time 24-h dietary recall. During the interview, trained interviewers asked the participants to report all food and beverages, including condiments and supplements, consumed the day before the interview. Energy, nutrient intake, and the energy supply ratio were further calculated according to the Chinese Food Composition Table (Yang, 2009) and compared with the Dietary Nutrient Reference Intake of Chinese lactating mothers (Chinese Nutrition Society, 2014). In addition, the food in the dietary survey was classified into 10 categories: cereals (rice, flour), soybean products, miscellaneous beans, nuts, livestock meat, freshwater products, edible oils, dairy, eggs, and seafood to calculate the food contribution rate of fat.

#### Breast milk sample collection

Each mother provided their breast milk before the infants’ fecal samples were collected. The nursing mother was required to feed the babies between 6 and 7 a.m. and empty both breasts. All the samples were collected between 9 and 11 a.m. The whole milk (including fore- and hindmilk) from one side of the breast was collected with a sterilized breast pump. Samples were gently mixed by inversion, then aliquoted, and immediately frozen at −80°C. All the samples were analyzed within 4 weeks.

#### Fecal samples

Mothers were instructed to collect 2 g of infant feces from the infant’s diaper with a sterilized collection tube, refrigerate it at home (at −12 to −14°C) for no more than 12 h, and transport it on an ice pack to the laboratory. Samples were stored at −80°C until analysis.

### Long-chain fatty acid analysis

Long-chain FAs were extracted from human milk using the method of Bligh and Dyer. Human milk was homogenized in 750 μL of chloroform: methanol 1:2 (v/v) with 10% deionized water and incubated at 4°C for 30 min. At the end of the incubation, 350 μL of deionized water and 250 μL of chloroform were added. The samples were then centrifuged, and the lower organic phase containing lipids was extracted into a clean tube. Lipid extracts were pooled into a single tube and dried in a SpeedVac in OH mode. Lipids were separated by normal-phase NP-HPLC, which was carried out using a Phenomenex Luna 3 μM silica column (internal diameter 150 × 2.0 mm) under the following conditions: mobile phase A (chloroform:methanol:ammonium hydroxide, 89.5:10:0.5) and mobile phase B (chloroform:methanol:ammonium hydroxide:water, 55:39:0.5:5.5). Long-chain FAs were analyzed using an Agilent 1,290 UPLC coupled to a triple quadrupole/ion trap mass spectrometer (6,500 Plus Qtrap; SCIEX).

### Extraction and bioinformatics analysis of the metagenome from samples

Genomic DNA was extracted with the QIAamp DNA Stool Mini Kit (QIAGEN, Hilden, Germany) and sequenced using the Illumina HiSeq sequencing platform, and clean data were obtained after pretreatment. Soap *de novo* assembly software was used for assembly analysis, and gene functional pathway and abundance analyses were performed based on the assembly results. Genes were compared with functional databases, and unigenes were compared with bacteria extracted from the NR (version: 2018.01) database of NCBI by DIAMOND software. Species annotation and abundance results were obtained, and the top 50 species with the largest relative abundance were selected. The R language tool was used to count the number of species, functions, or genes shared or unique in multiple samples and to construct abundance spectra at the genus and species levels. QIIME (quantitative insights into microbial ecology) software was used to analyze the diversity of the intestinal flora, including Chao, ACE, Shannon, Simpson, etc. The results were displayed in a box chart, and the *t*-test and the Kruskal–Wilcox nonparametric test were used to analyze the significance of differences between groups. The mixOmics package in the R language was used to analyze the microbial community data by partial least squares discrimination analysis (PLS-DA). Differences in lipid species between groups were detected by the rank-sum test, and the species with significant biomarkers between groups were screened by LDA (linear discriminant analysis). Kyoto Encyclopedia of Genes and Genomes (KEGG) is a comprehensive database for systematic analysis of gene function, linkage genomic information, and functional information, which is composed of several sub-databases, such as the pathways database. Most of the known pathways, including nucleotide metabolism and organic biodegradation, can be found in the pathway database and are divided into three levels according to the pathway and functional information on genes, proteins, and related biochemical compounds. In this study, KEGG function analysis was used to explore the differences in the expression of functional genes and pathways of infants’ intestinal microorganisms under different breast milk fatty acid composition levels. Kruskal–Wallis rank-sum test was used to compare the KEGG functional levels of different FA groups in breast milk and to further screen for relevant differences in KEGG ORTHOLOGY (KO) and corresponding to the relevant EC. STAMP software (2.1.3) was used for visualization.

### Statistical analysis

The data were analyzed with SAS version 9.3 (SAS Institute, Inc., Cary, NC, United States). Continuous variables are statistically described by mean ± standard deviation (SD) and median (P25, P75), and categorical variables are described by proportion or composition ratio. The long-chain FA levels in breast milk were grouped into tertiles as high, medium, or low. Spearman’s rank correlation was used to analyze the correlation between the content of long-chain FAs in breast milk and the 24-h dietary intake of lactating mothers, including fat intake and the energy supply ratio, food intake, and the proportional contribution of each food to fat intake.

## Results

### Basic characteristics of 56 pairs of mothers and infants

The socio-demographic characteristics, lifestyle, and health-related indicators of the 56 pairs of mothers and infants are shown in Table 1. The average maternal age was 29.9 ± 3.9 years. The average birth weight of the infants was 3,325 (3,000, 3,513) g. Among them, the majority of babies were born by vaginal delivery, and male infants accounted for 55.4%.

**Table 1:** Basic characteristics of the 56 pairs of mother and infant.

Characteristics		Description
**Mother**		
Age (years)		29.9 ± 3.9
Gestational week		38.8 ± 0.9
Household monthly per-capita income^a^ (RMB: yuan)	<3,000	7 (13.0)
	3,000–4,000	47 (87.0)
Parity	First	30 (53.5)
	Second	22 (39.3)
Current BMI (kg/m^2^)		22.6 ± 6.6
**Infant**		
Gender	Male	31 (55.4)
	Female	25 (44.6)
Mode of delivery^b^	Vaginal delivery	45 (81.8)
	Cesarean section	10 (18.2)
Birth weight (g)		3,325 (3,000,3,513)

Continuous variables were presented as the Mean ± SD or median (P25, P75) according to normality of data. Categorical variables were presented as N (%). a means that the income of 54 people was available. b means that the delivery mode of 55 people was available.

### Dietary intake of lactating mothers

The dietary intake of the participating mothers is presented in Table 2. Compared with the dietary nutrient reference intake for Chinese lactating mothers (Chinese Nutrition Society, 2014), 72.1% of lactating mothers’ total energy intake was lower than the dietary energy requirement (2,300 kcal per day), and 55.7% of the mothers ingested more protein than the recommended 80 g per day.

**Table 2:** Dietary intake of lactating mothers.

	Dietary intake^1^		Comparison with dietary recommendation	
	Mean ± SD	P50 (P25,P75)	<Reference value	≥Reference value
Total energy (kcal)	2063.7 ± 703.0	1974.1 (1667.4,2371.3)	44 (72.1)	17 (27.9)
Protein (g)	83.3 ± 34.4	81.8 (59.1,99.8)	27 (44.3)	34 (55.7)
Fat (g)	79.2 ± 38.9	67.3 (53.9,96.3)	–	–
Carbohydrate (g)	256.5 ± 121.4	237.8 (176,288.9)	–	–
Cholesterol (mg)	696.7 ± 632.3	518.6 (43,1232.9)	–	–

^1^Continuous variables are statistically described by Mean ± SD and Median (P25, P75), classified variables are described by N (proportion).

### Long-chain fatty acids in breast milk

We determined 15 kinds of long-chain FAs in human milk by UPLC-MS. The long-chain FA content is shown in Table 3. The total content of long-chain FAs was 500.4 ± 321.9 mg/l, of which the most abundant was oleic acid (C18:2), followed by linoleic acid (C18:1), and palmitic acid (C16:0).

**Table 3:** Contents of long-chain fatty acids in breast milk.

Fatty acids type	Fatty acids	Mean ± SD	P50 (P25, P75)
long-chain FAs (mg/L)	C14:0	15.5 ± 14.5	10.7 (6.8, 17.3)
	C14:1	0.6 ± 0.7	0.4 (0.3, 0.7)
	C15:0	0.6 ± 0.6	0.4 (0.3, 0.6)
	C16:0	52.6 ± 30.8	43.9 (33.4, 63.0)
	C16:1	4.8 ± 3.0	4.0 (3.1, 5.7)
	C18:0	34.2 ± 9.8	33.8 (27.3, 38.3)
	C18:1	59.3 ± 41.0	46.6 (35.6, 71.2)
	C18:2	276.5 ± 186.8	208.7 (169.9, 321.5)
	C18:3	32.8 ± 30.8	24.1 (14.0, 39.5)
	C20:3	1.1 ± 0.9	0.7 (0.6, 1.3)
	C20:4	9.7 ± 8.7	6.0 (4.8, 10.9)
	C20:5	1.1 ± 1.5	0.7 (0.4, 1.4)
	C22:4	0.9 ± 0.9	0.7 (0.4, 1.1)
	C22:5	4.4 ± 3.6	3.3 (2.1, 5.2)
	C22:6	6.2 ± 4.6	5.2 (3.1, 8.4)
	Total	500.4 ± 321.9	400.6 (304.0, 589.5)

### Correlation between the dietary intake of lactating mothers and the long-chain fatty acid content in breast milk

We first explored the correlation of breast milk long-chain FA content with total fat intake and the fat energy contribution ratio of lactating mothers (Supplementary Table S1) and found that the breast milk long-chain FA content had no significant correlation with fat intake and the fat energy contribution ratio.

**Figure 1:**
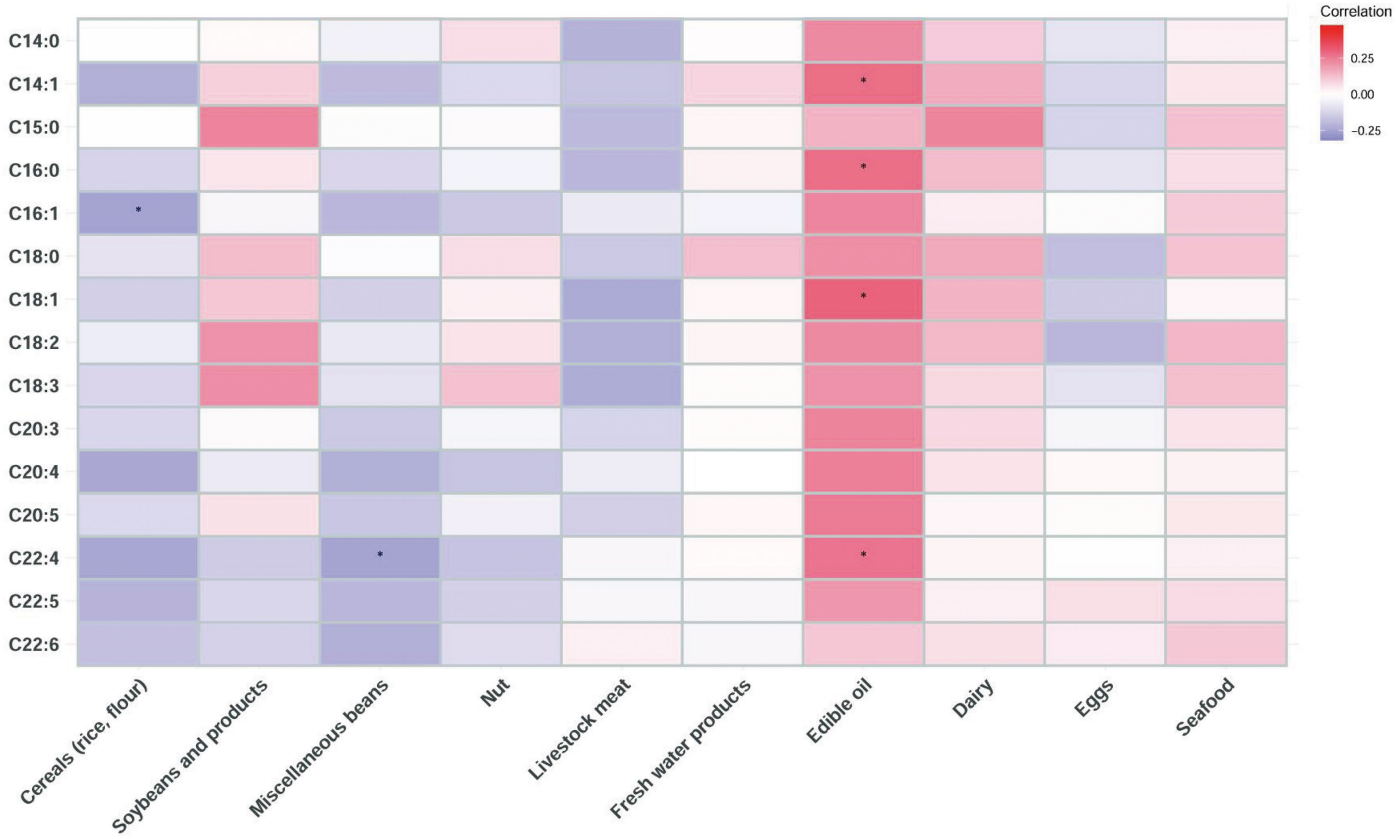
Correlation heatmap of the long-chain FA content in human milk and the food contribution rate of fat intake of lactating mothers based on Spearman analysis (* indicates *p* < 0.05).

The correlation between the long-chain FA content in breast milk and the food contribution rate of fat intake was further explored (Figure 1). The results showed that the contents of C14:1, C16:0, C18:1, and C22:4 were positively correlated with the fat contribution rate of edible oil. The C22:4 content of breast milk was negatively correlated with the fat contribution rate of miscellaneous beans. The C16:1 content of breast milk was negatively correlated with the fat contribution rate of cereals (rice and flour).

### Association of breast milk long-chain fatty acids with infants’ intestinal microbiota

We grouped the long-chain FA levels in breast milk into high, medium, and low tertiles and further analyzed the association of the diversity and composition of infants’ intestinal microbiota with different breast milk long-chain FA levels. For the alpha diversity index, there were no significant between-group differences in the Chao, ACE, or Shannon and Simpson indexes (Supplementary Figure S1).

PLS-DA analysis showed that the contents of different long-chain FAs in breast milk, including C14:0, C15:0, C16:1, C18:0, C18:1, C18:2, C20:3, C20:4, C20:5, C22:4, C22:5, and C22:6, were significantly associated with the overall composition of the infant intestinal microbiota (Supplementary Figure S2).

To further analyze the impact of milk long-chain FAs on the specific composition of the infant intestinal microbiota, we analyzed the above association at the genus and species levels, respectively (Supplementary Figure S3). The results showed that the infant intestinal microbiota was mainly composed of *Bifidobacteria, Escherichia, Lactobacillus, Lacticaseibacillus,* and *Bacteroides* at the genus level.

**Figure 2:**
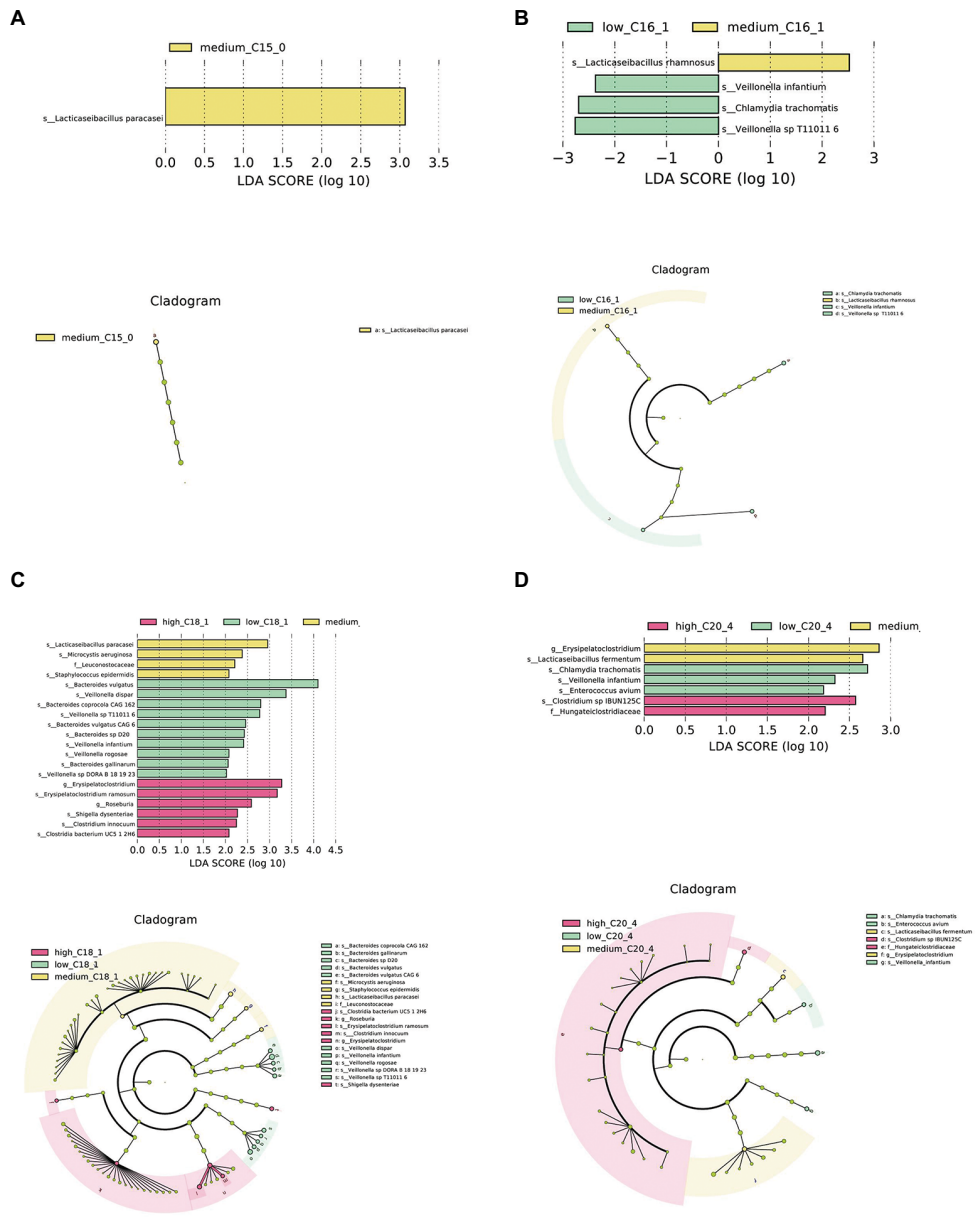
Biomarkers of differences between different FA content groups in human milk. **(A–D)** LEfSe analysis of C15:0, C16:1, C18:1, and C20:4 groups, respectively. Taxonomic cladogram obtained from LEfSe and linear discriminant analysis (LDA) of these groups. Biomarker taxa are highlighted with colored circles and shaded areas. Each circle’s diameter reflects the abundance of those taxa in the community, using a cutoff value of ≥2.0.

Further, we conducted LEfSe analysis on the intestinal microbiota of infants to determine significant biomarkers of long-chain FAs in human milk. It was found that infants in the medium C15:0 tertile had a relatively high abundance of *Lacticaseibacillus paracasei* (Figure 2A). A significantly different microbial biomarker was also found for C16:1, in which the medium tertile had a relatively high abundance of *Lacticaseibacillus rhamnosus* (Figure 2B). According to the analysis of C18:1, the medium tertile had a relatively higher abundance of *L. paracasei* (Figure 2C). The medium C20:4 tertile had a relatively high abundance of *Lacticaseibacillus fermentum* (Figure 2D).

### Functional pathway

**Figure 3:**
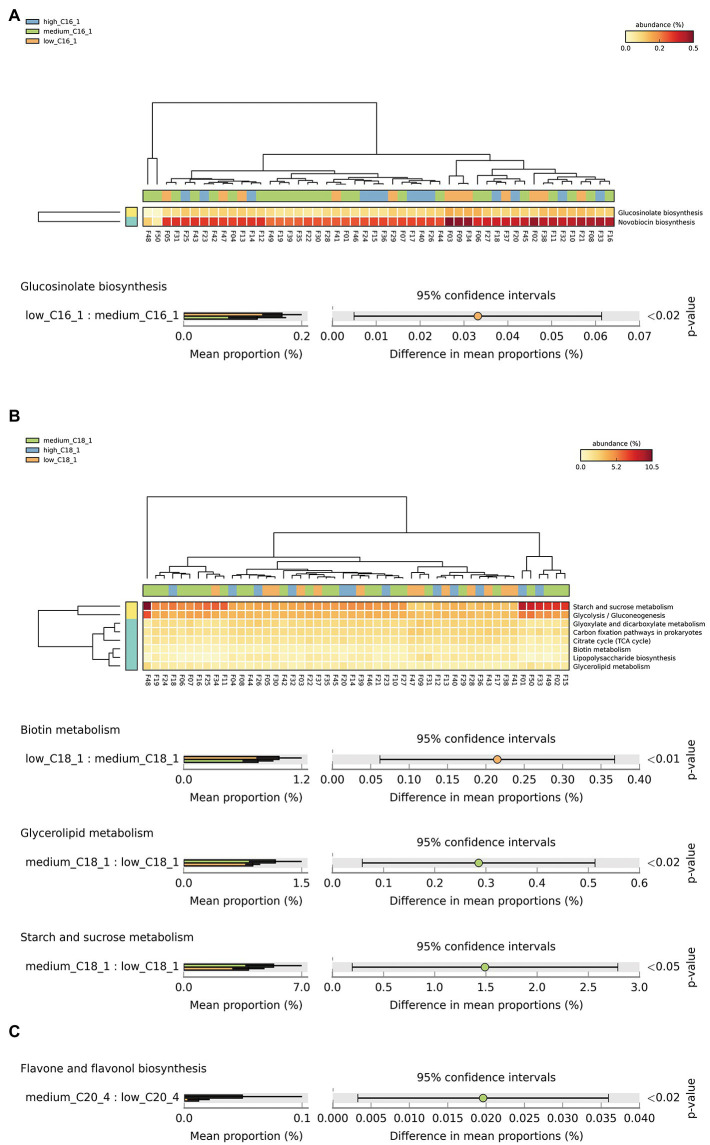
Analysis of specific long-chain FAs and KEGG tertiary metabolic pathway abundance levels in human milk with different FA contents. (**A–C)** The KEGG tertiary enrichment and differential pathways of breast milk C16:1, C18:1, and C20:4, respectively, in different content groups (orange, green, and blue indicate low, medium, and high content, respectively). Kruskal–Wallis rank sum test was used to compare the KEGG functional levels of different FA groups in breast milk (orange, green and blue indicate low, medium, and high content, respectively). The color transition from yellow to red indicates that the abundance of this metabolic pathway gradually increases in the sample.

Based on the above analysis of significant biomarkers of intestinal flora at the species level, we screened the key long-chain FAs in breast milk for their effects on the intestinal flora of infants, including C15:0, C16:1, C18:1, and C20:4. We then conducted a functional correlation study. We classified the metabolic pathways at the functional level using KEGG pathway analysis, which resulted in 6 first-level, 45 s-level, and 422 third-level metabolic pathways. Among them, the functional expression of the infant intestinal flora belonged to the first-level pathways metabolism, environmental information processing, cellular processes, genetic information processing, human diseases, and organic systems. To analyze the overall effects of the above key long-chain FAs in human milk on the corresponding infant metabolic pathways, we attempted to analyze 200 tertiary metabolic pathways under 17 KEGG secondary metabolic pathway branches, including carbohydrate metabolism, amino acid metabolism, cofactors and vitamins, lipid metabolism, circulatory system, digestive system, endocrine system, excretory system, immune system, etc. The results showed significant differences in Glucosinolate biosynthesis between C16:1 consumption levels (Figure 3A). There were significant differences in biotin, glycerolipid, starch, and sucrose metabolism between C18:1 consumption levels (Figure 3B). There was a significant difference in flavone and flavonol biosynthesis between C20:4 consumption levels (Figure 3C).

We further searched the KEGG Orthology (KO) corresponding to the above-mentioned differential metabolic pathways in the KEGG database.

**Figure 4:**
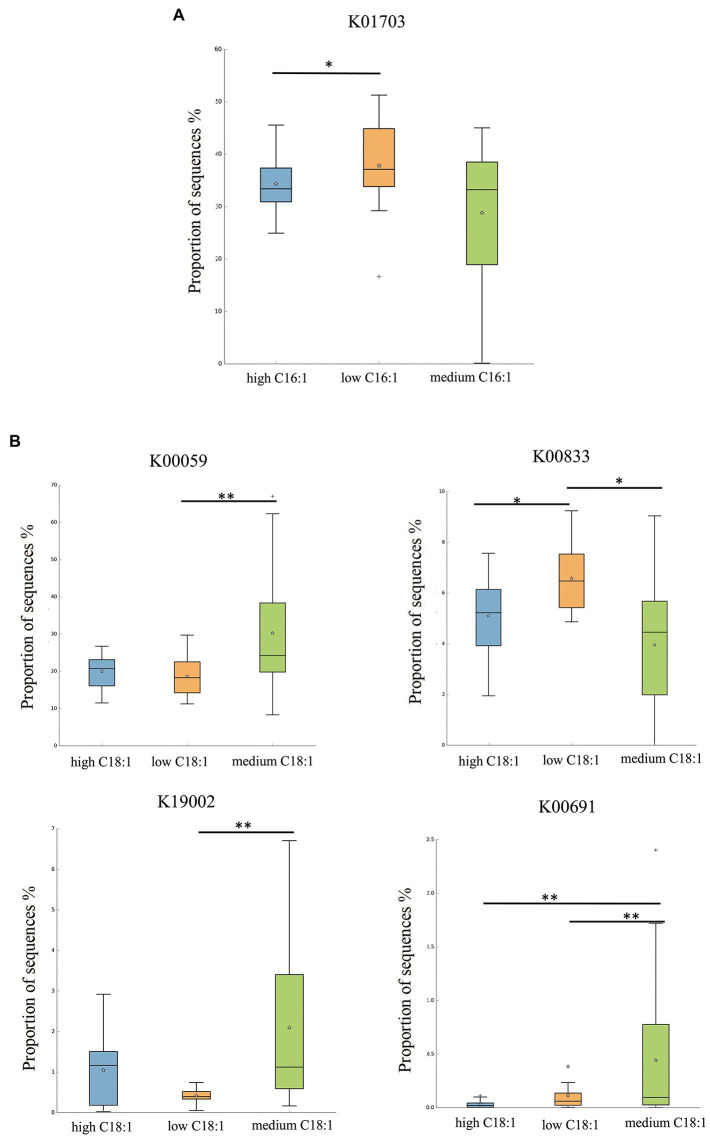
KO analysis of significant differences for certain breast milk long-chain FAs. **(A,B)** represent the KO analysis of breast milk C16:1, and C18:1, respectively, of different content groups, and the results are shown in the box diagram (orange, green, and blue indicate low, medium, and high content, respectively). * indicates *p* < 0.05, ** indicates *p* < 0.01.

For C16:1, the most significant corresponding signal pathway was Glucosinolate biosynthesis, which involved 21 related KO reported in the KO database. When these KO were compared to the 10,016 KO annotated with the flora data from this study, it was found that 18 were under the glucosinolate biosynthesis pathway. Kruskal–Wallis nonparametric test was used to compare the relative KO abundances between C16:1 content groups. Only the functional relative abundances of K01703 were significantly different between groups (Figure 4A).

For C18:1, the most significant corresponding signal pathways were biotin, glycerolipid, starch, and sucrose metabolism, which involved 24, 90, and 106 related KO reported in the KO database of KEGG, respectively. When these KO were compared to 10,016 KO annotated with the flora data from this study, it was found that 24 KO were annotated under the biotin metabolism pathway, 78 KO under the glycerolipid metabolism pathway, and 101 KO under the starch and sucrose metabolism pathway. Kruskal–Wallis nonparametric test was used to compare the relative KO abundances between C18:1 content groups. We found significant between-group differences in the functional relative abundances of K00833, K00059, K19002, and K00691 (Figure 4B). There was no significant difference in the KO involved in different signal pathways between C15:0 and C20:4 content groups.

Based on the KEGG database, the EC names and functions corresponding to the above differences in KO were obtained. It can be seen that K01703 to 3-isopropylmalate/(R)-2-methylmalate dehydratase large subunit [EC:4.2.1.33 4.2.1.35], K00833 to adenosylmethionine-8-amino-7-oxononanoate aminotransferase [EC:2.6.1.62], K00059 to 3-oxoacyl-[acyl-carrier protein] reductase [EC:1.1.1.100], K19002 to 1,2-diacylglycerol 3-alpha-glucosyltransferase [EC:2.4.1.337], and K00691 to maltose phosphorylase [EC:2.4.1.8].

## Discussion

In this study, a correlation analysis was performed to clarify the specific factors that affect breast milk long-chain FAs in the diets of nursing mothers and the effect of different contents of long-chain FAs in breast milk on the intestinal microbiota of infants. To the best of our knowledge, we were the first to report, based on KEGG pathway analysis, that different levels of long-chain FAs in breast milk were significantly correlated with the presence of particular microbial species in the infant intestine, especially *Lactobacillus* and *Lacticaseibacillus,* related to pathways of biotin metabolism, glycerolipid metabolism, and starch and sucrose metabolism.

### Long-chain fatty acids in breast milk and their association with diet

Long-chain FAs in breast milk mainly originate in the liver. After dietary and body-derived FAs are transformed into very low-density lipoprotein particles, they are transported to the breast through the circulatory system for synthesis (Kong et al., 2021). This study first explored the correlation between the diets of lactating mothers and the long-chain FA content of their breast milk and showed that the levels of different FAs in breast milk were related to the intake of certain types of food. Edible oils were the most significant contributors, and their C22:4, C18:1, C16:0, and C14:1 contents were positively correlated with those of breast milk. Our results were similar to those of previous studies. Wang et al. reported that the intake of dietary oil was positively correlated with the content of n-6 PUFA and C18:2 in colostrum (Wang L. et al., 2020). At present, the main edible oil consumed in China is vegetable oil, and the common commercial edible oils include soybean oil, rapeseed oil, peanut oil, cottonseed oil, etc. (Zhang et al., 2021). As an important source of the human body’s daily intake of fat and FAs, edible oil is rich in fatty acids, mainly including C16:0, C18:0, C18:1, and C18:2, of which C18:1 accounts for 75–85% of the total FAs in edible oil (Zhang et al., 2021).

It should be noted that the most abundant long-chain FAs in breast milk are C18:2, followed by C18:1 and C16:0. There are few reports on the correlation between breast milk C16:0, C18:1, and dietary intake. This study supports and extends previous studies by demonstrating that there was a significant contribution of maternal dietary edible oil to the dominant fatty acid content in breast milk. In addition, we should mention that oil intake continues to increase in the Chinese population, already exceeding the recommended amount (25–30 g; Chinese Nutrition Society, 2014). Chinese lactating mother oil intake could be as high as 36–38 g (Zhao et al., 2021). Besides contributing massive amounts of certain FAs to breast milk, it should not be ignored that the excessive intake of oil may pose a risk to women of developing a series of chronic diseases, such as obesity, fatty liver, and cardiovascular diseases (Wallace, 2019). Since the C18:2, C18:1, and C16:0 in breast milk are the major source of energy for infants and have specific biological functions, whether or not high maternal edible oil intake could influence infant growth is a particularly interesting research question.

Besides edible oils, other oil-containing foods were also found to affect the breast milk FA content; for example, previous studies found that C18:2, C18:3, and C22:6 in human milk were more positively correlated with a diet rich in nuts and seeds, eggs, and meat; meanwhile, meat product consumption frequencies were negatively correlated with C18:2 and C18:3 (Aumeistere et al., 2019). Tian et al. (2019) divided 274 lactating mothers into four groups according to their dietary patterns and showed that there were significant differences in the composition of SFA, PUFA, and n-6 PUFA in breast milk corresponding to the different dietary patterns, the characteristics of which mainly depended on meat, mushroom, and marine product intake; soybean product, nut, and dairy intake; vegetable and fruit intake; and grain/potato, bean, and egg intake, respectively. In the current study, we observed inverse associations of C16:1 with cereals and of C22:4 with miscellaneous beans. C16:1 and C22:4 are usually abundant in seafood and meat, respectively, and have a limited content in plant-based foods (Jagodic et al., 2020). A diet high in cereals and miscellaneous beans may indicate a lower proportion of animal food intake, where a negative association occurred.

The current study did not find that diet could affect the DHA level in breast milk. DHA is less abundant in human milk but was reported to be tightly associated with the maternal diet. Quinn and Kuzawa (2012) found that milk DHA showed a positive, dose–response relationship with maternal fish consumption. An Iranian study showed that mothers who consumed two servings of fish per week during pregnancy had higher DHA levels in their breast milk than those who did not eat fish (Beheshteh et al., 2012). The inconsistent results may be explained by the relatively small intake of seafood in the traditional Chinese diet (Hu et al., 2014). At the same time, population variations may exist because the genetic factors may also affect the synthesis and expression of long-chain FAs in liver cells. The factors that affect breast milk DHA content should be further determined because DHA is one of the most important functional long-chain FAs in the early development of the neural and visual systems of infants.

### The association of breast milk fatty acids and the infant intestinal flora

This study found that the composition of the intestinal microflora in infants could be significantly affected by maternal FAs. According to the results of diversity analysis, multiple types of FAs affect the overall composition, distribution, and status of the intestinal flora in infants.

According to a genus-level analysis, the main constituent microorganisms of the infant intestinal flora are *Bifidobacteria, Escherichia, Lactobacillus, Lacticaseibacillus,* and *Bacteria*. This result was consistent with those of Ma et al. (2022), who reported on the main constituent bacteria in the intestinal tracts of infants and abroad in the early stage of growth and development. These bacteria were believed to play an important role in the maturation of the immune system and the intestinal barrier in early life of infants (Yan et al., 2021).

Most previous studies used 16S rRNA amplicon sequencing technology to explore the effects of long-chain FAs in breast milk on the infant intestinal flora at the genus level. For example, Jiang et al. explored the effects of the 10 most abundant sn-2 FAs in Chinese breast milk on the infant intestinal microbial composition and found a significant association between sn-2 FAs (including C14:0, C18:0, C16:0, C20:4, and C22:6) in milk and the infant gut microbiota (such as *Bacteroides, Veillonella, Streptococcus,* and *Clostridium)* (Jiang et al., 2018); however, the regulatory effects of FAs on certain species could not be determined and the effects on species level were still unknown.

To explore the potential prebiotic-like properties of breast milk, we further explored significant differences in the microbial composition among different long-chain FA levels and identified relevant intestinal flora biomarkers. At the species level, we found that the level of breast milk long-chain FAs mainly regulates the abundances of *L. rhamnosus, L. fermentum,* and *L. paracasei*. Different long-chain FA groups have different specific targeted regulatory flora, for example, *L. rhamnosus* was higher in the medium C16:1 content group, *L. paracasei* was significantly higher in the medium C18:1 content group than in the low and high groups, and *L. fermentum* was higher in the medium C20:4 content group. The higher probiotic abundance only found in the medium-content group may be caused by random effects due to the limited sample size in this study. We inferred there might be a non-linear association between FAs and the abundance of certain microbes in the infant gut. According to the results mentioned above, excessive intake of edible oils contributes to a high content of C16:1, C18:1, and C22:4 in breast milk. As a previous study revealed, excessive FA intake can increase the ratio of steroids from bile to estrogen and change the composition of the intestinal flora, which suggests that a reasonable intake of a certain amount of FAs can promote the proliferation of beneficial bacteria and play a role in maintaining health (Cui, 2020). However, there has been no such study conducted on the infant population. Whether the current phenomenon is caused by random effects or there is a U-shaped correlation between FAs and the intestinal flora should be further confirmed.

Perhaps not coincidentally, the above three kinds of probiotics, which are easily affected by maternal breast milk FAs, have certain protective functions in the body. Considering the potential functions of the infant gut flora, we further analyzed the gene expression differences and metabolic pathways of different breast milk FA groups through the KEGG pathway and revealed that the main gene expression pathways of different long-chain FAs differed. The results showed that breast milk C18:1 related to K00833 and K00059 in the biotin metabolism pathway, corresponding to adenosylmethionine-8-amino-7-oxononanoate aminotransferase and 3-oxoacyl-[acyl-carrier protein] reductase, respectively. These two enzymes were responsible for the important cellular metabolic processes of methylation and acetylation in biotin metabolism. Biotin belongs to the water-soluble B vitamins, which participate in the processes of methylation and acetylation in the form of epigenetics and play a key role in regulating the expression of genes related to carbohydrate, protein, and lipid metabolism (León-Del-Río, 2019). To the best of our knowledge, our study is the first to reveal that the content of C18:1 in breast milk may affect biotin metabolism in infants, which needs to be verified by subsequent studies. Moreover, this study also revealed that a certain amount of long-chain FAs in breast milk may have a potential role in regulating infant lipid and carbohydrate metabolism. C16:1 regulated K01703 in the glucosinolate biosynthesis pathway, corresponding to the large subunit of 3-isopropylmalate/(R)-2-methylmalate dehydratase. C18:1 regulated K19002 in the glycerolipid metabolism pathway, corresponding to 1,2-diacylglycerol 3-alpha-glucosyltransferase, and K00691 in the starch and sucrose metabolism pathway, corresponding to maltose phosphorylase, respectively. There are limited data regarding the large subunit of 3-isopropylmalate/−2-methylmalate dehydratase. 1,2-diacylglycerol-3-alpha-glucosyltransferase is a triacylglycerol synthetic enzyme, which may be related to fat and glycolipid metabolism, however, its biological function is still not clear. It should be noted that a large number of studies have confirmed the significant lipid-lowering effects of *L. fermentum* and *L. rhamnosus* (Wang T. et al., 2020; Molina-Tijeras et al., 2021). Evidence from ours and previous studies suggest that maternal breast milk lipids, such as C18:1, could further regulate infant lipid metabolism and that this effect might be mediated by shaping of the infant gut microbiota. Another enzyme linked with C18:1 in this study is Maltose phosphorylase, a glycoside hydrolase family of 65 enzymes, reversibly phosphorylates maltose. It catalyzes the reversible phosphorolysis of maltose to D-glucose and β-D-glucose 1-phosphate (β-Glc1P), which is then absorbed and utilized by the body. The enzyme is part of operons that are involved in maltose/malto-oligosaccharide metabolism (Gao et al., 2019). Many studies have reported that *Lactobacillus* spp., such as *Lactobacillus brevis* and *Lactobacillus acidophilus*, contain genes and structures encoding maltose phosphorylase (Egloff et al., 2001). The current results suggest that C18:1 in breast milk may regulate this enzyme through specific intestinal microbiota, and then play a regulatory role in carbohydrate metabolic pathway.

To briefly summarize the results of KEGG pathway analysis, we revealed the potential role of breast milk long-chain FAs in regulating infant metabolism, *via* regulation of the intestinal flora. Further epidemiological studies on infants or *in vivo* studies will be helpful to illustrate and determine the multiple functions of long-chain FAs in early life and their related mechanisms.

### Strengths and limitations

The strength of this study lies in its use of metagenome sequencing, which can identify specific intestinal bacteria at the species level that could be affected by FAs. It also adopted the method of maternal–infant pairs to explore the role of the maternal diet in infant health. In addition, we extended previous studies by not only demonstrating the effects of specific FAs on infants’ intestinal flora but also showing the comprehensive contribution of breast milk FAs to the infant microbiota and identified the maternal diet as the predisposing factor.

Three limitations should be addressed in our study. Firstly, the sample size was limited. As such, future research with a representative large sample size is required to confirm these results. Secondly, during the 24-h dietary recall, many participants were unable to clarify the specific kind of cooking edible oil or reported using mixed oils, which, to some extent, restricted our further analysis of FA sources. In addition, there is still a lack of data regarding the FA contents of many foods in the Chinese Food Composition Table, which hinders direct evaluation of the associations between the specific FA composition of breast milk and that of the maternal diet. Thirdly, this study is a cross-sectional study, and causality could not be further confirmed. Moreover, the long-term effect of the maternal diet on breast milk FAs and that of FAs on the infant gut flora should be examined in future studies.

## Conclusion

Based on metagenomic and UPLC-MS analyses, the current results suggest that the maternal diet, especially its content of edible oil, is highly associated with breast milk long-chain FAs and could further affect the abundance of specific intestinal microbes in infants, such as *L. rhamnosus* and *L. paracasei.* In addition, the current study revealed a potential pathway of certain types of long-chain FAs that could regulate the expression of enzymes, which correspond to the potential regulatory functions of biotin, carbohydrate, and lipid metabolism. These findings may illustrate the potential bond between maternal and infant metabolism, even after birth. Further prospective and maternal–infant paired studies are needed to clarify the pathways relating dietary intake, breast milk lipids, the infant gut flora, and infant health and to explore the corresponding mechanisms.

## Data availability statement

The data presented in the study are deposited in the NCBI repository, accession number PRJNA858926. The data has been released, which can be found at: https://www.ncbi.nlm.nih.gov/bioproject/PRJNA858926/.

## Ethics statement

All participants gave written informed consent following the Declaration of Helsinki. The protocol was approved by the Research Center for Public Health, Tsinghua University (NO.THUSM/PHREC/2021–003). Written informed consent to participate in this study was provided by the participants’ legal guardian/next of kin.

## Author contributions

MX contributed to conceptualization, methodology, investigation, validation, formal analysis, and writing—original draft. XN, XM, and HL contributed to conceptualization, methodology, and investigation. TS and W-HL contributed to conceptualization and supervision. WH and AZ contributed to writing, reviewing, and editing the manuscript. All authors contributed to the article and approved the submitted version.

## Conflict of interest

XM, HL, TS, W-HL, and WH were employed by Inner Mongolia Dairy Technology Research Institute Co., Ltd. and Inner Mongolia Yili Industrial Group Co., Ltd.

The remaining authors declare that the research was conducted in the absence of any commercial or financial relationships that could be construed as a potential conflict of interest.

## Publisher’s note

All claims expressed in this article are solely those of the authors and do not necessarily represent those of their affiliated organizations, or those of the publisher, the editors and the reviewers. Any product that may be evaluated in this article, or claim that may be made by its manufacturer, is not guaranteed or endorsed by the publisher.
