# Supplementary material for: Maternal diet associated with infants’ intestinal microbiota mediated by predominant long-chain fatty acid in breast milk
Source: Front Microbiol. 2023 Jan 6;13:1004175. doi: 10.3389/fmicb.2022.1004175 (PMC9852834; doi:10.3389/fmicb.2022.1004175)
Supplement: Supplementary file 1 [file Data_Sheet_1.PDF]

SUPPLEMENTARY MATERIAL

C14:0

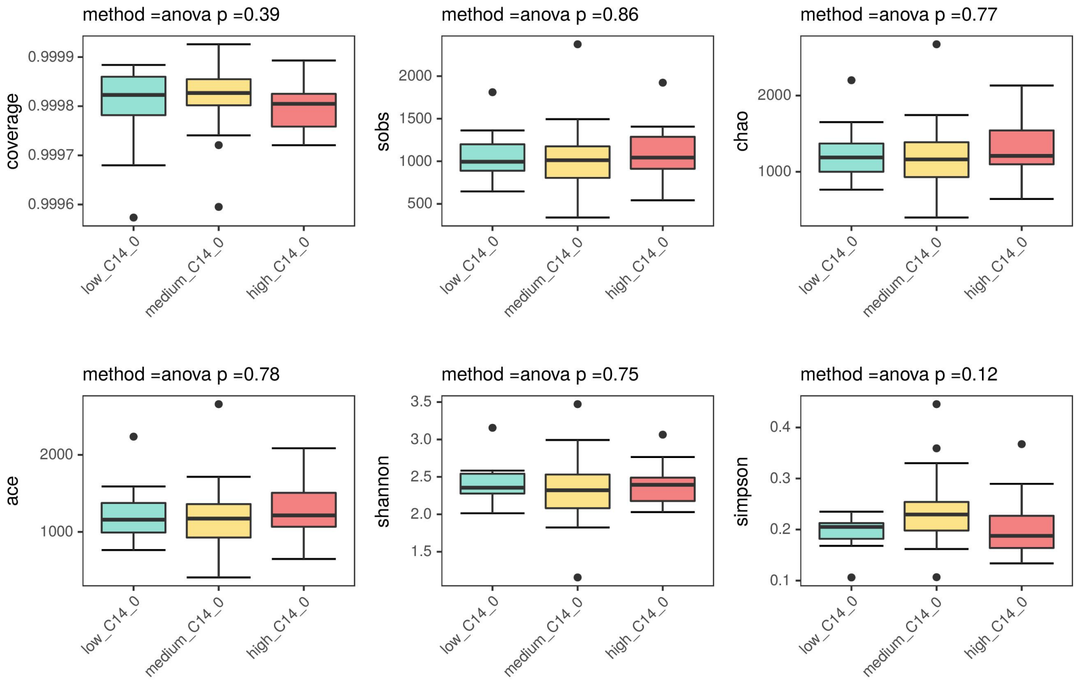

C14:1

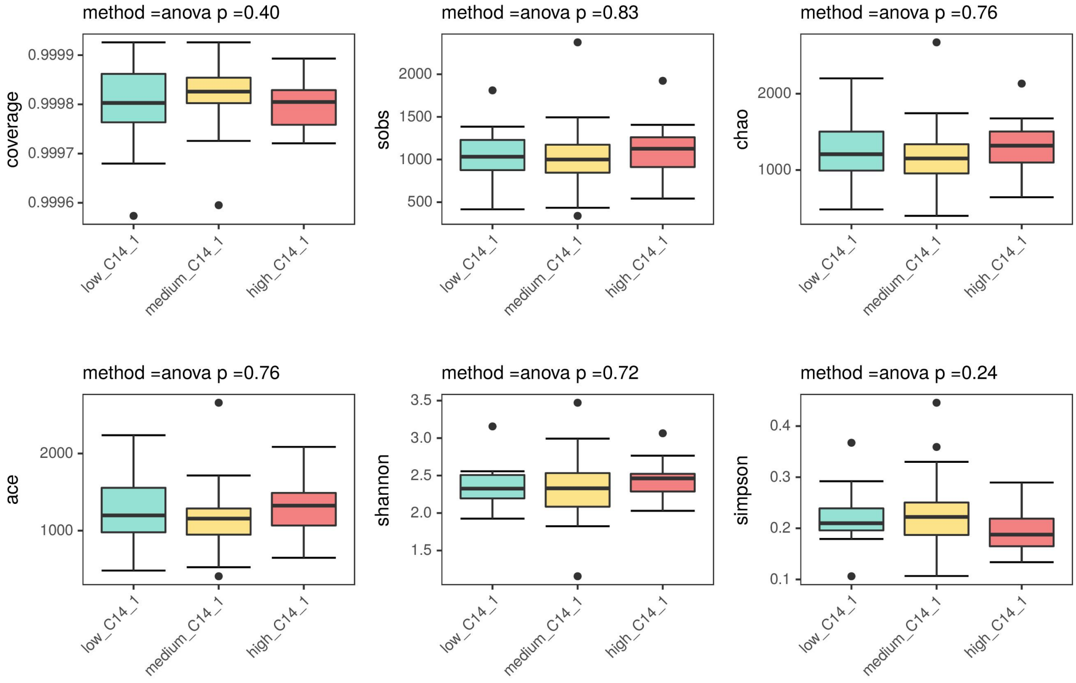

C15:0

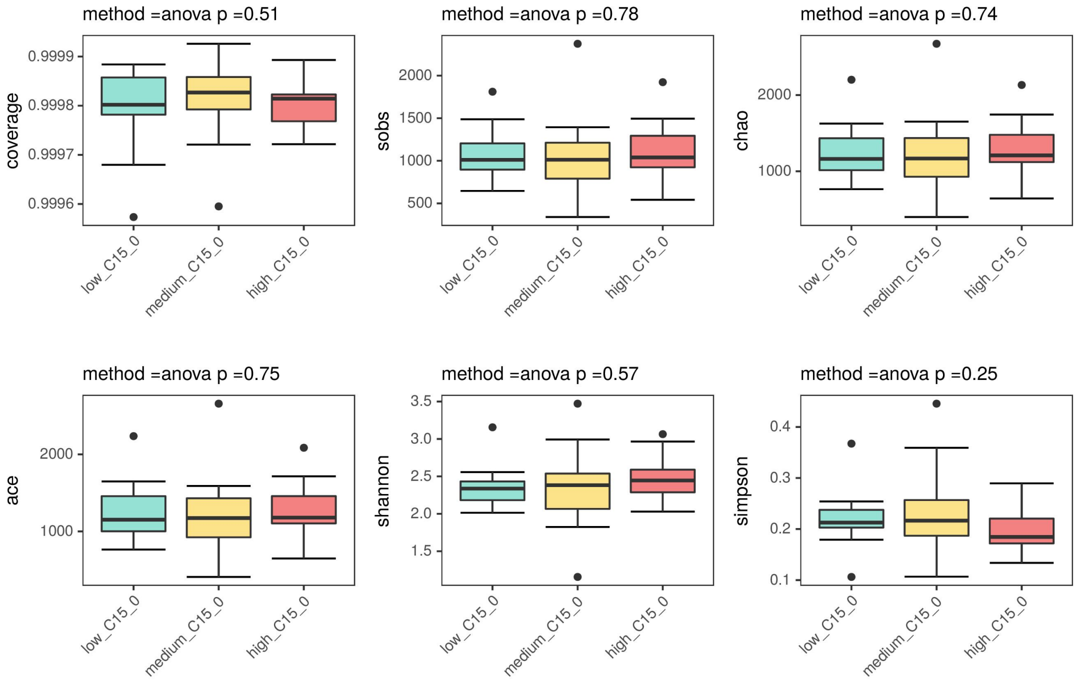
method =anova p =0.51
coverage
0.9999
0.9998
0.9997
0.9996
low_C15_0
medium_C15_0
high_C15_0
method =anova p =0.78
sobs
2000
1500
1000
500
low_C15_0
medium_C15_0
high_C15_0
method =anova p =0.74
chao
2000
1000
low_C15_0
medium_C15_0
high_C15_0
method =anova p =0.75
ace
2000
1000
low_C15_0
medium_C15_0
high_C15_0
method =anova p =0.57
shannon
3.5
3.0
2.5
2.0
1.5
low_C15_0
medium_C15_0
high_C15_0
method =anova p =0.25
simpson
0.4
0.3
0.2
0.1
low_C15_0
medium_C15_0
high_C15_0

C16:0

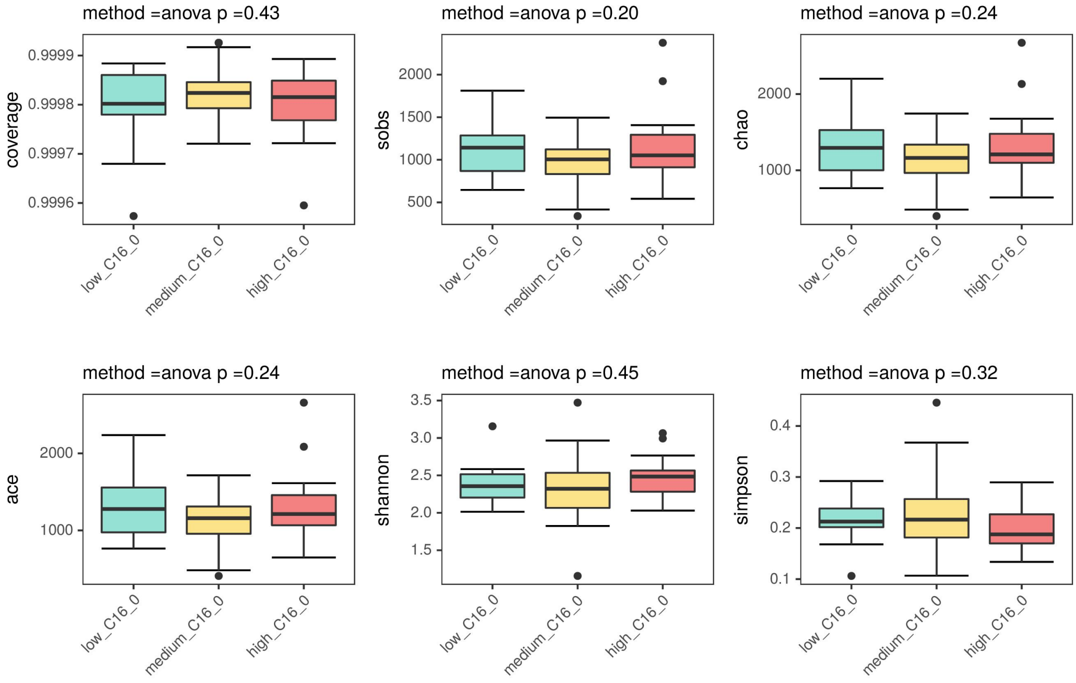
method =anova p =0.43
coverage
0.9999
0.9998
0.9997
0.9996
low_C16_0
medium_C16_0
high_C16_0
method =anova p =0.20
sobs
2000
1500
1000
500
low_C16_0
medium_C16_0
high_C16_0
method =anova p =0.24
chao
2000
1000
low_C16_0
medium_C16_0
high_C16_0
method =anova p =0.24
ace
2000
1000
low_C16_0
medium_C16_0
high_C16_0
method =anova p =0.45
shannon
3.5
3.0
2.5
2.0
1.5
low_C16_0
medium_C16_0
high_C16_0
method =anova p =0.32
simpson
0.4
0.3
0.2
0.1
low_C16_0
medium_C16_0
high_C16_0

C16:1

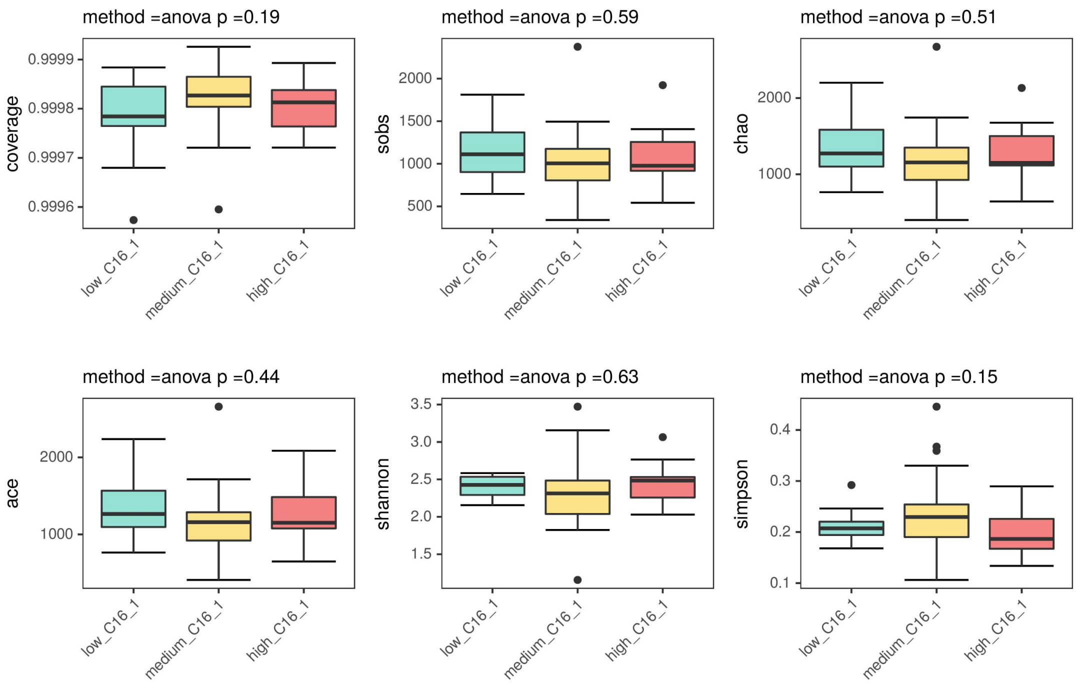
method =anova p =0.19
coverage
0.9999
0.9998
0.9997
0.9996
low_C16_1
medium_C16_1
high_C16_1
method =anova p =0.59
sobs
2000
1500
1000
500
method =anova p =0.51
chao
2000
1000
method =anova p =0.44
ace
2000
1000
method =anova p =0.63
shannon
3.5
3.0
2.5
2.0
1.5
method =anova p =0.15
simpson
0.4
0.3
0.2
0.1

C18:0

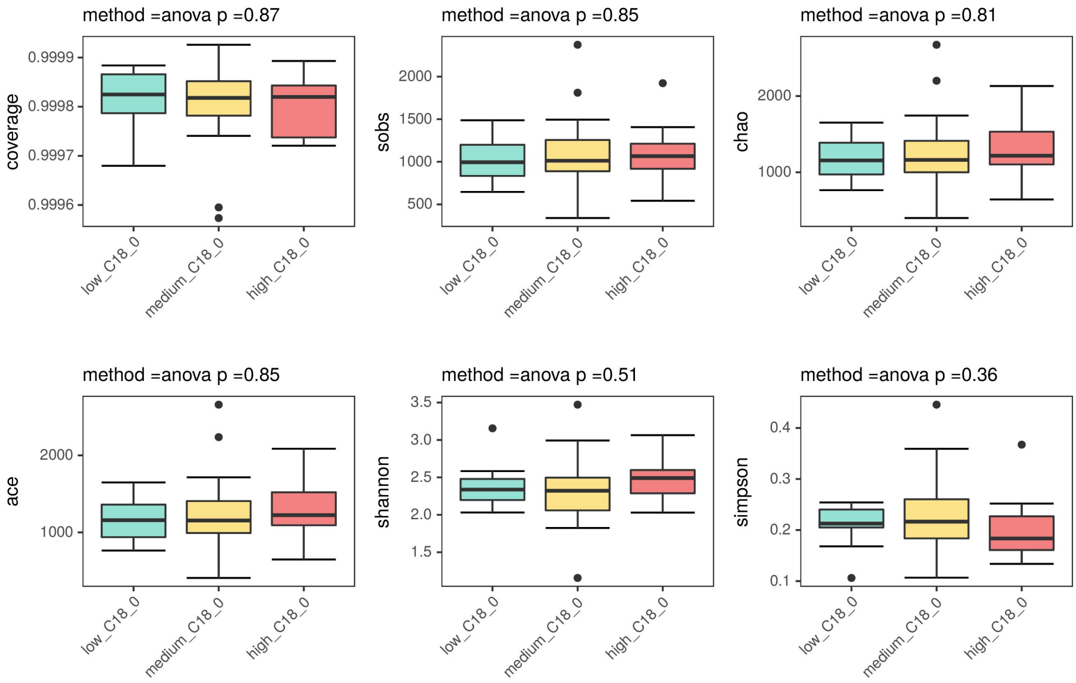
method =anova p =0.87
coverage
0.9999
0.9998
0.9997
0.9996
low_C18_0
medium_C18_0
high_C18_0
method =anova p =0.85
sobs
2000
1500
1000
500
method =anova p =0.81
chao
2000
1000
method =anova p =0.85
ace
2000
1000
method =anova p =0.51
shannon
3.5
3.0
2.5
2.0
1.5
method =anova p =0.36
simpson
0.4
0.3
0.2
0.1

C18:1

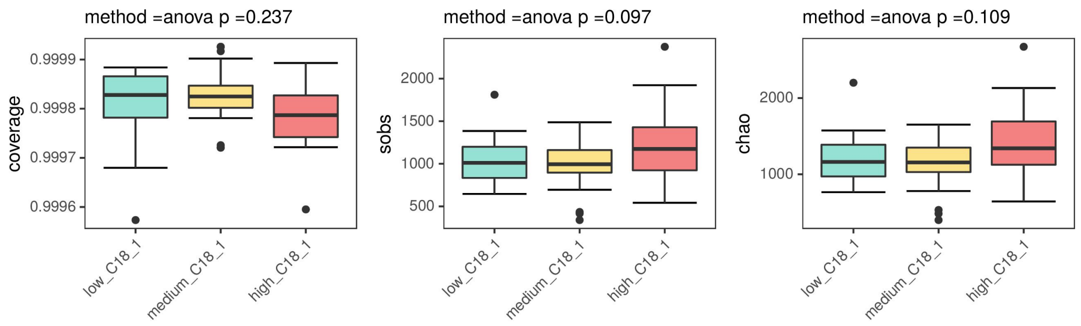
method =anova p =0.237
method =anova p =0.097
method =anova p =0.109
method =anova p =0.118
method =anova p =0.179
method =anova p =0.190
coverage
sobs
chao
ace
shannon
simpson
low_C18_1
medium_C18_1
high_C18_1

C18:2

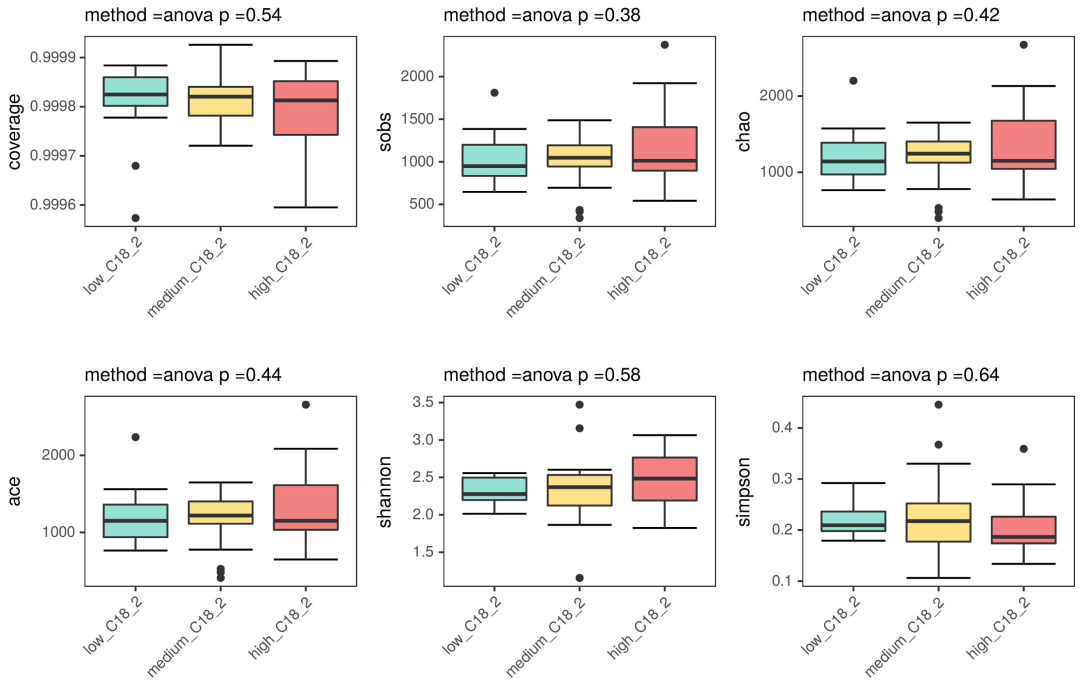
method =anova p =0.54
method =anova p =0.38
method =anova p =0.42
method =anova p =0.44
method =anova p =0.58
method =anova p =0.64
coverage
sobs
chao
ace
shannon
simpson
low_C18_2
medium_C18_2
high_C18_2

C18:3

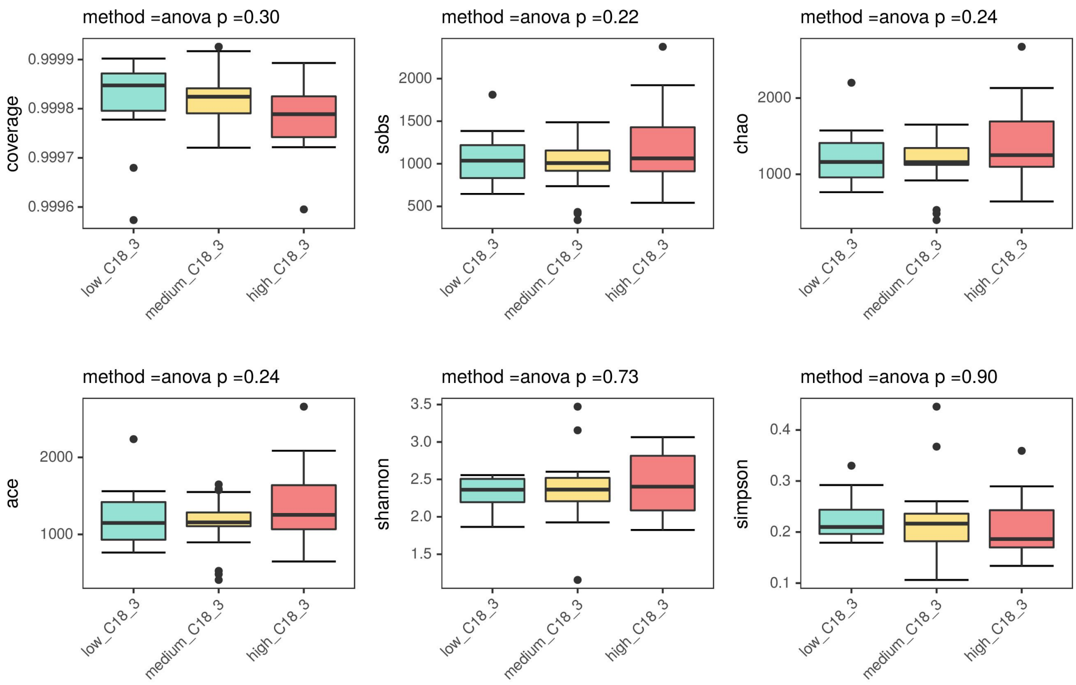
method =anova p =0.30
method =anova p =0.22
method =anova p =0.24
method =anova p =0.24
method =anova p =0.73
method =anova p =0.90
coverage
sobs
chao
ace
shannon
simpson
low_C18_3
medium_C18_3
high_C18_3

C20:3

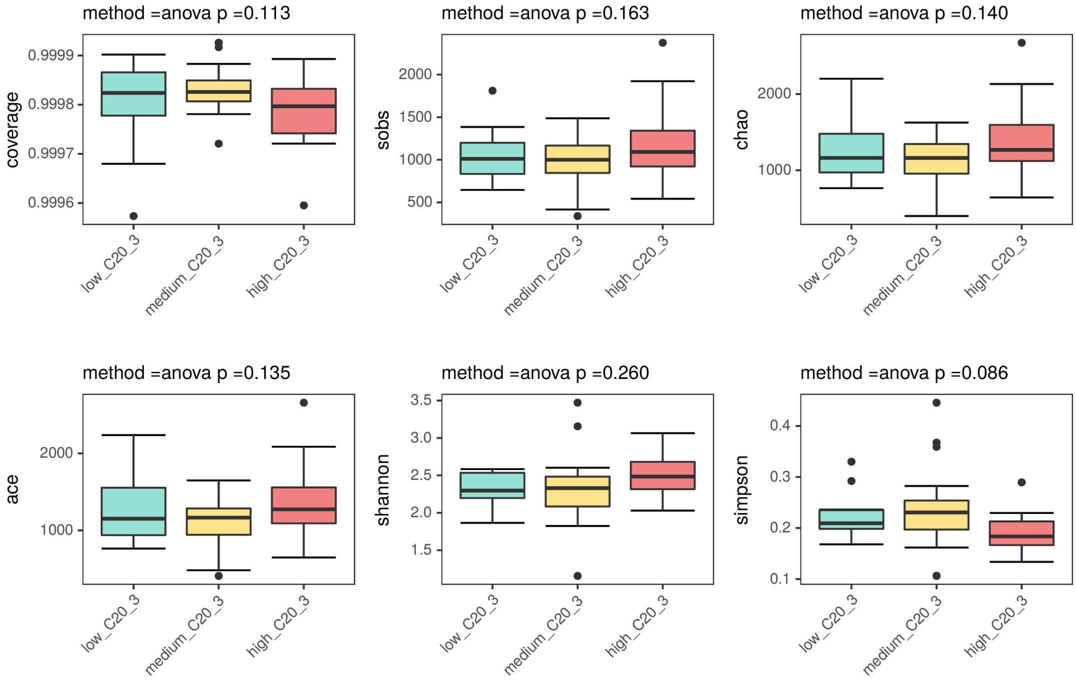
method =anova p =0.113
method =anova p =0.163
method =anova p =0.140
method =anova p =0.135
method =anova p =0.260
method =anova p =0.086
coverage
sobs
chao
ace
shannon
simpson
low_C20_3
medium_C20_3
high_C20_3

C20:4

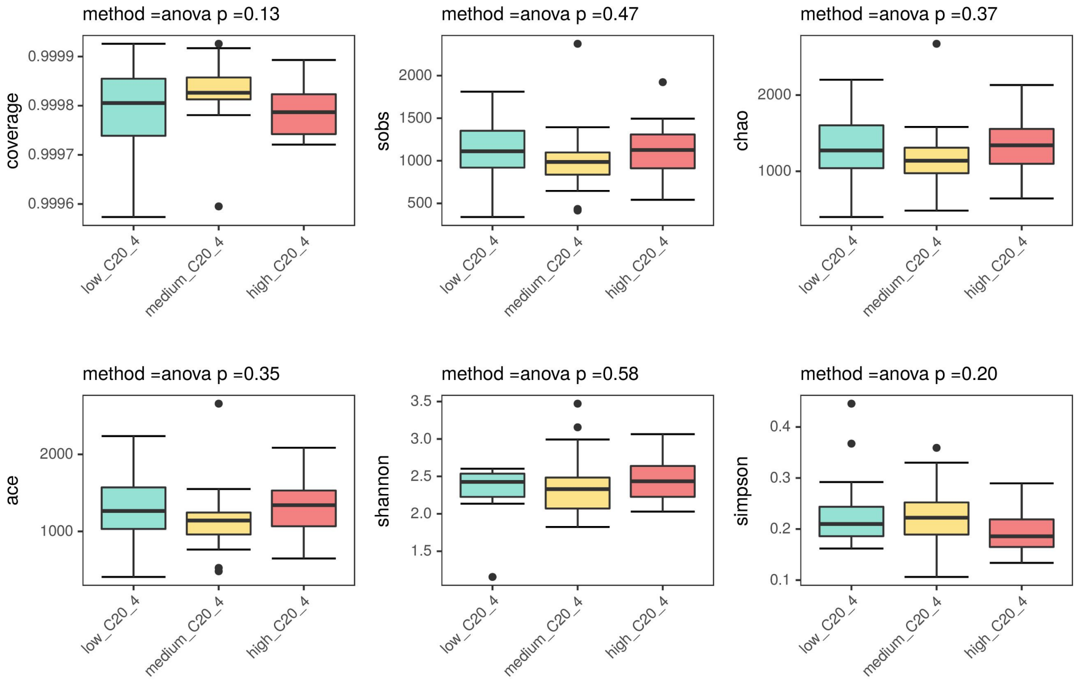
method =anova p =0.13
coverage
0.9999
0.9998
0.9997
0.9996
low_C20_4
medium_C20_4
high_C20_4
method =anova p =0.47
sobs
2000
1500
1000
500
method =anova p =0.37
chao
2000
1000
method =anova p =0.35
ace
2000
1000
method =anova p =0.58
shannon
3.5
3.0
2.5
2.0
1.5
method =anova p =0.20
simpson
0.4
0.3
0.2
0.1

C20:5

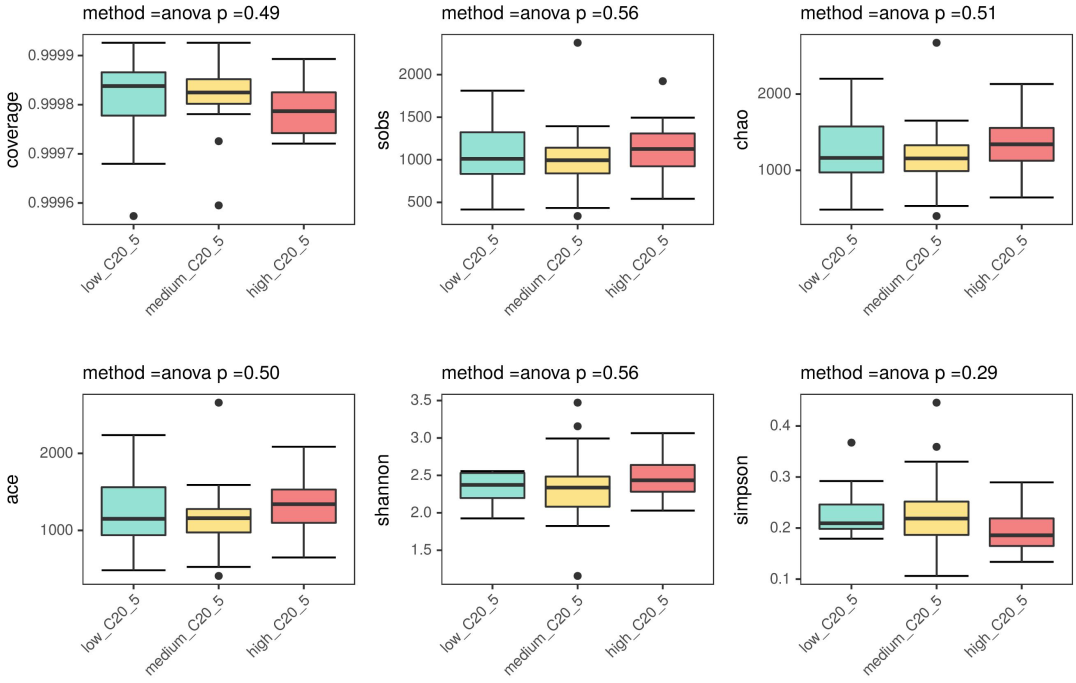
method =anova p =0.49
coverage
0.9999
0.9998
0.9997
0.9996
low_C20_5
medium_C20_5
high_C20_5
method =anova p =0.56
sobs
2000
1500
1000
500
method =anova p =0.51
chao
2000
1000
method =anova p =0.50
ace
2000
1000
method =anova p =0.56
shannon
3.5
3.0
2.5
2.0
1.5
method =anova p =0.29
simpson
0.4
0.3
0.2
0.1

C22:4

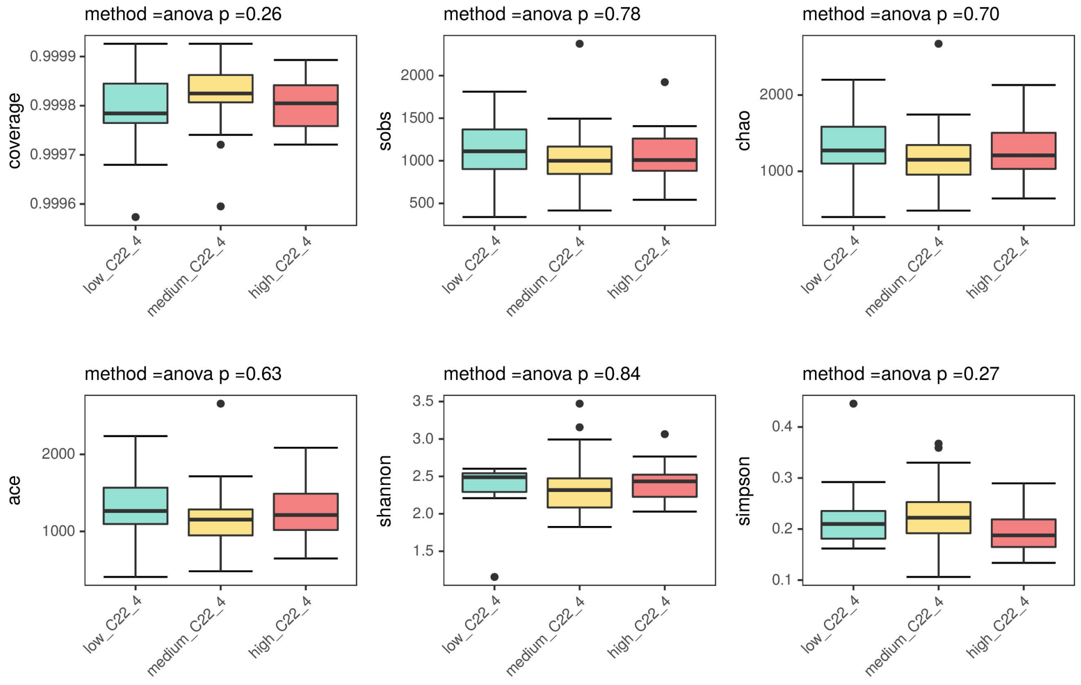
method =anova p =0.26
method =anova p =0.78
method =anova p =0.70
method =anova p =0.63
method =anova p =0.84
method =anova p =0.27
coverage
sobs
chao
ace
shannon
simpson
low_C22_4
medium_C22_4
high_C22_4

C22:5

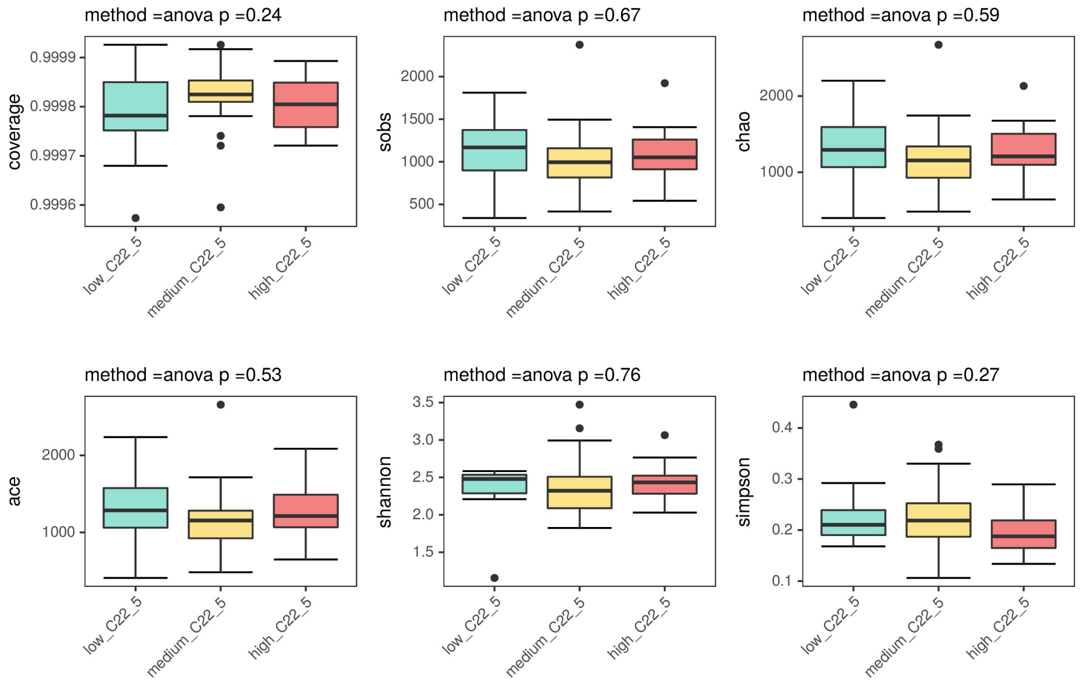
method =anova p =0.24
method =anova p =0.67
method =anova p =0.59
method =anova p =0.53
method =anova p =0.76
method =anova p =0.27
coverage
sobs
chao
ace
shannon
simpson
low_C22_5
medium_C22_5
high_C22_5

C22:6

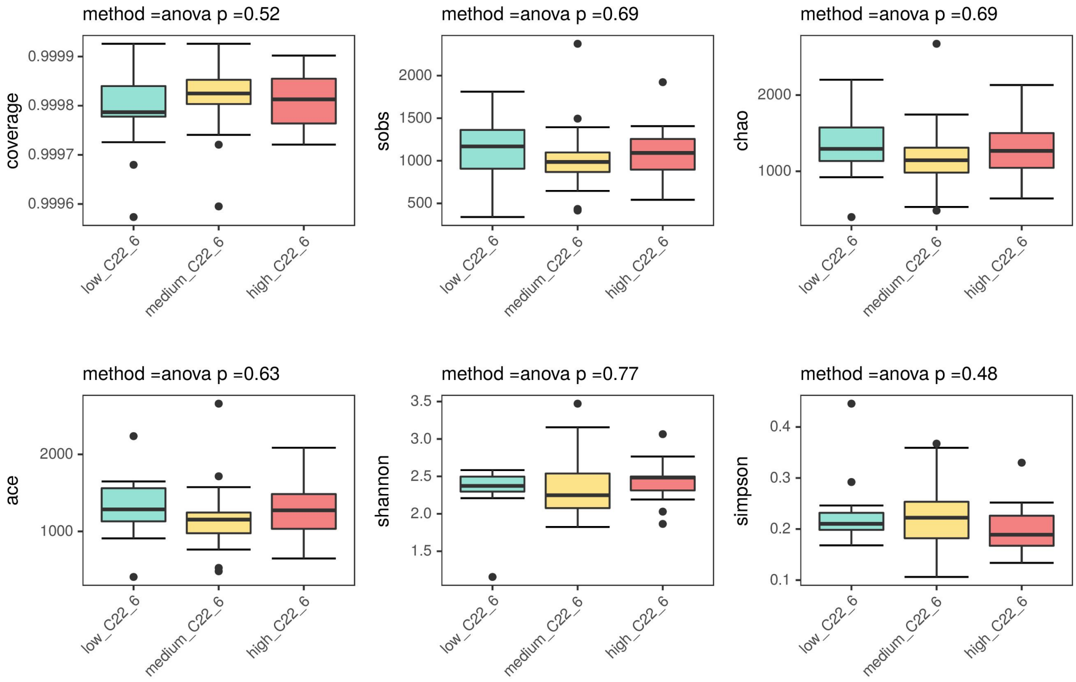

Figure S1. The Chao1 index, ACE, and Shannon and Simpson indexes for the long-chain FAs in different groups of breast milk.

C14:0

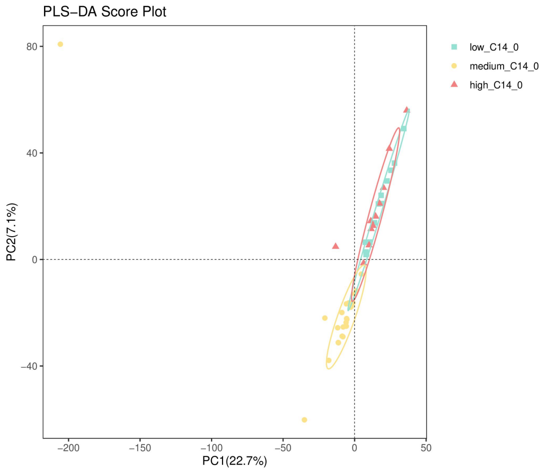

C14:1

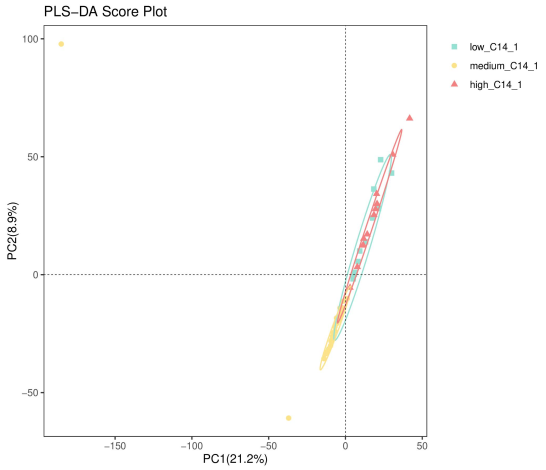

C15:0

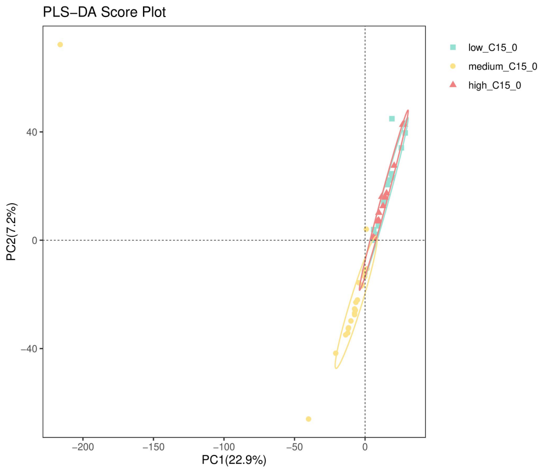

C16:0

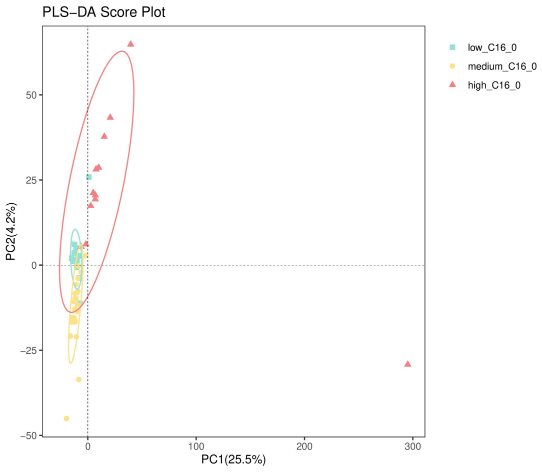

C16:1

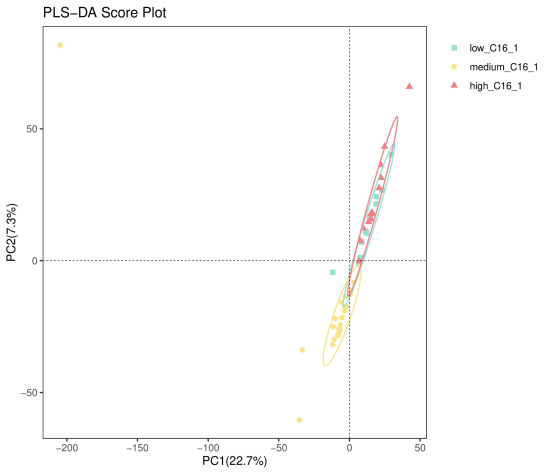

C18:0

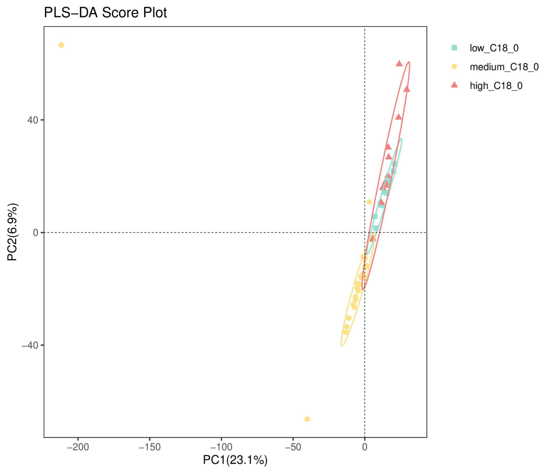

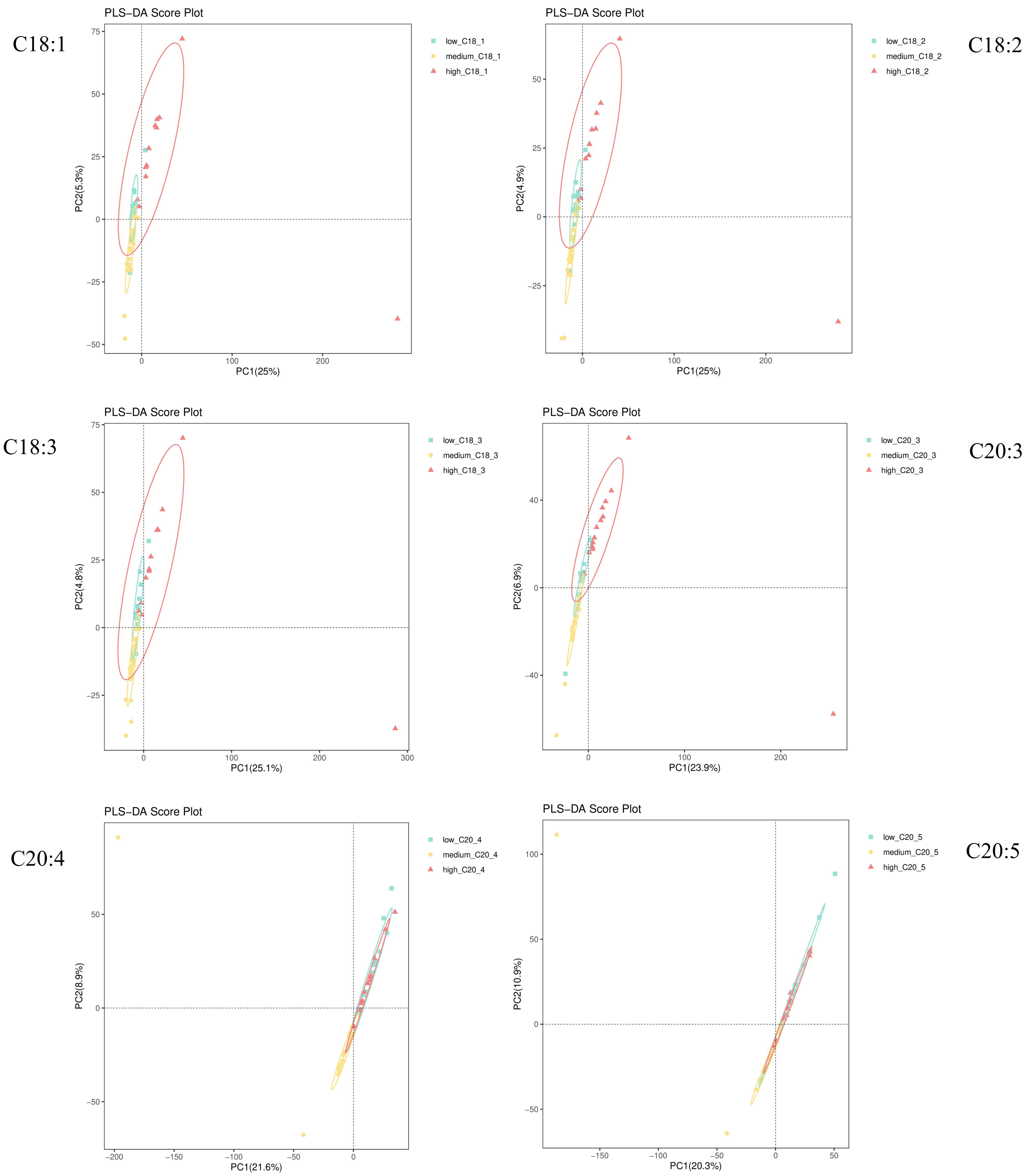
C18:1
PLS−DA Score Plot
low_C18_1
medium_C18_1
high_C18_1
PC1(25%)
PC2(5.3%)
C18:2
PLS−DA Score Plot
low_C18_2
medium_C18_2
high_C18_2
PC1(25%)
PC2(4.9%)
C18:3
PLS−DA Score Plot
low_C18_3
medium_C18_3
high_C18_3
PC1(25.1%)
PC2(4.8%)
C20:3
PLS−DA Score Plot
low_C20_3
medium_C20_3
high_C20_3
PC1(23.9%)
PC2(6.9%)
C20:4
PLS−DA Score Plot
low_C20_4
medium_C20_4
high_C20_4
PC1(21.6%)
PC2(8.9%)
C20:5
PLS−DA Score Plot
low_C20_5
medium_C20_5
high_C20_5
PC1(20.3%)
PC2(10.9%)

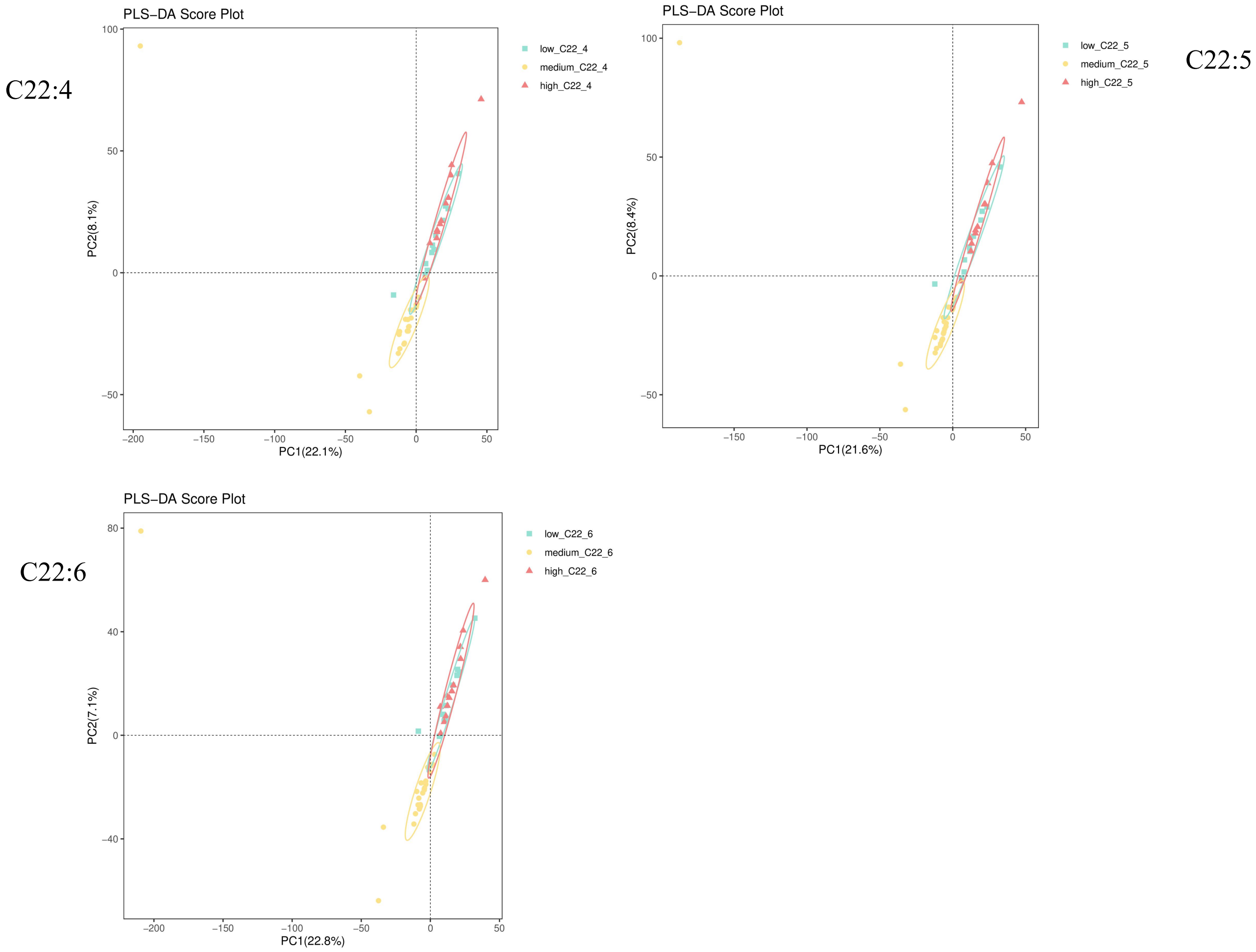

Figure S2. Overall distribution of infant intestinal microorganisms corresponding to the long-chain FAs in different groups of breast milk based on PLS-DA analysis.

FAs genus species

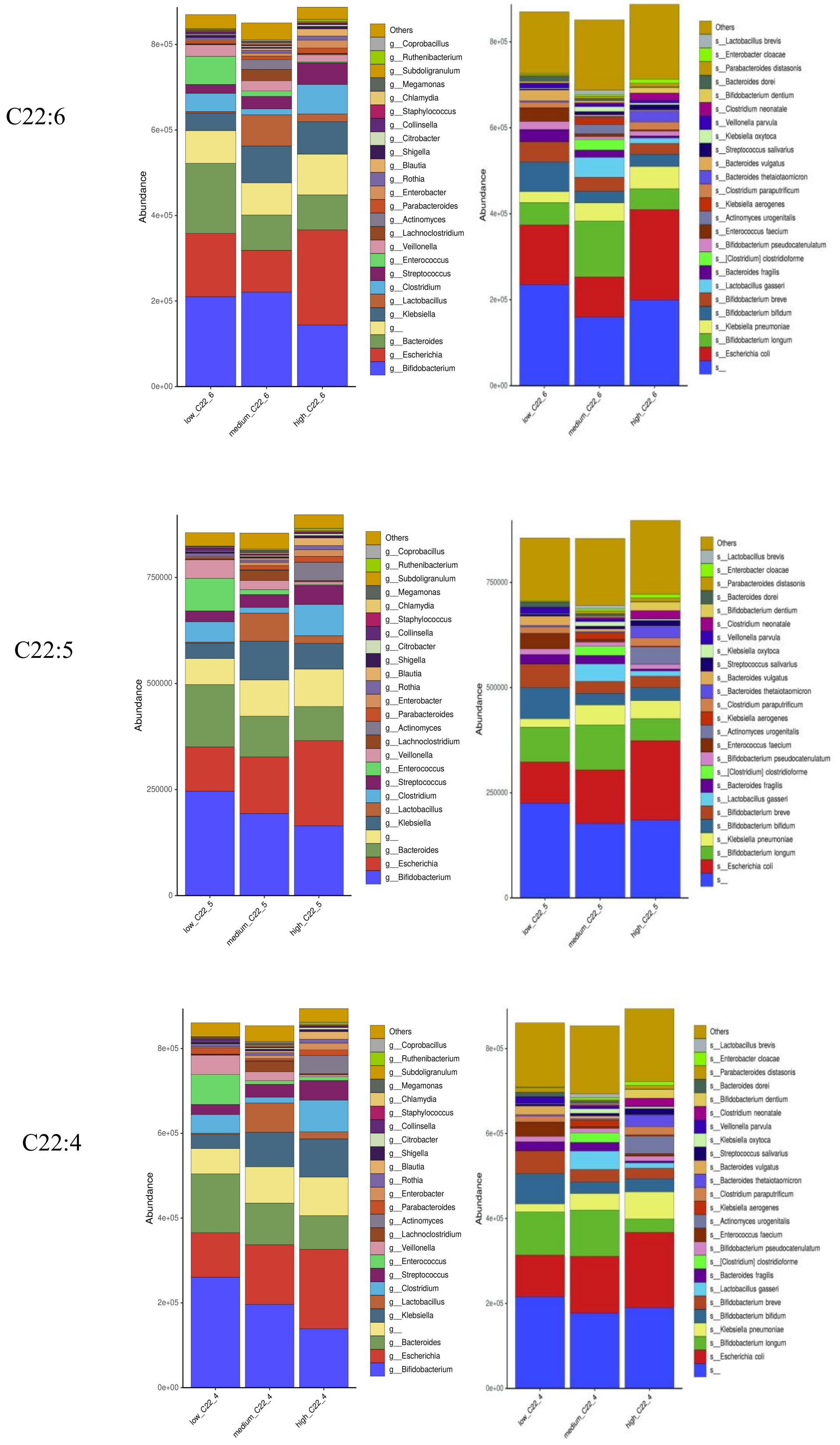

C20:5

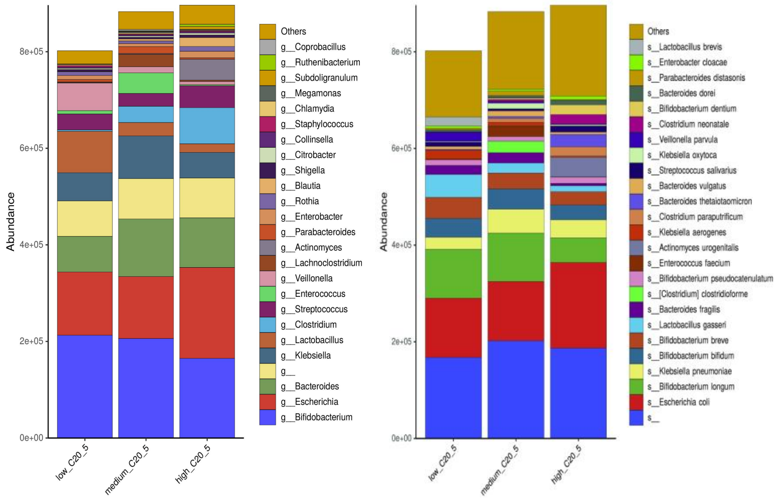

C20:4

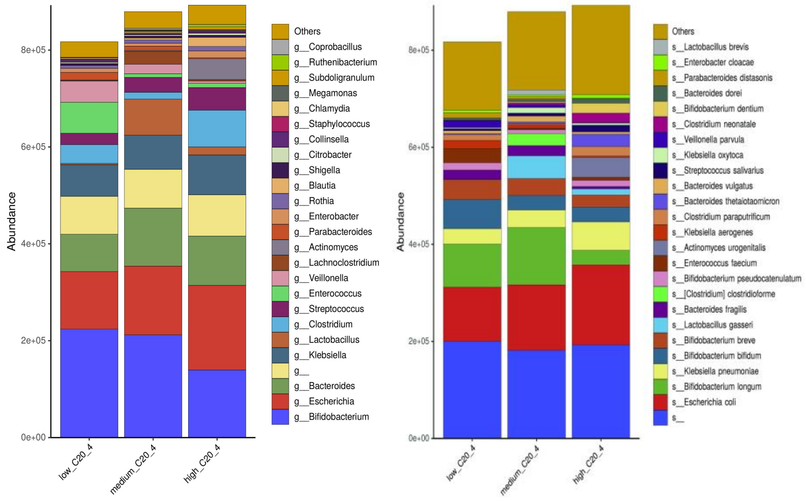

C20:3

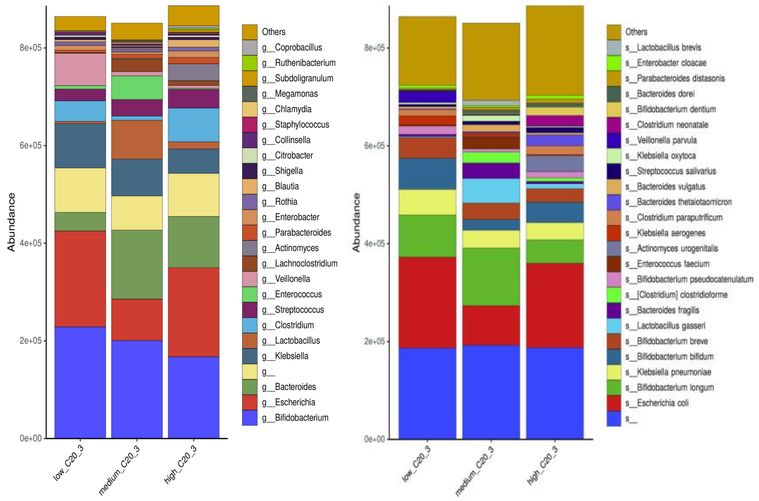

C18:3

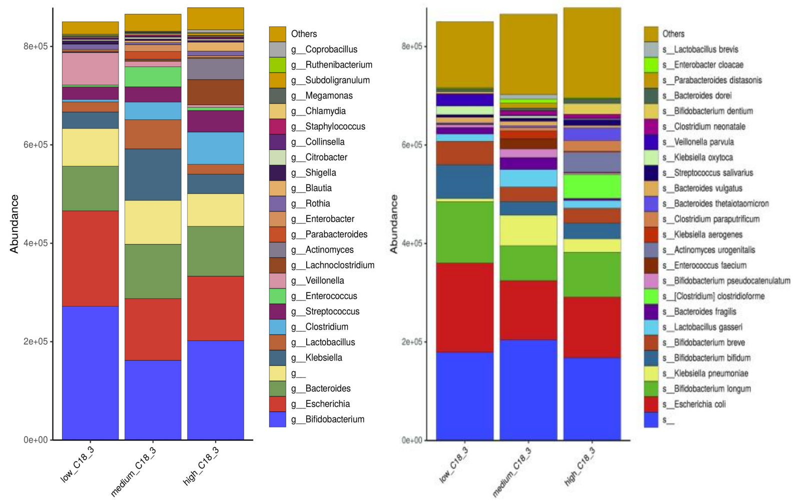

C18:2

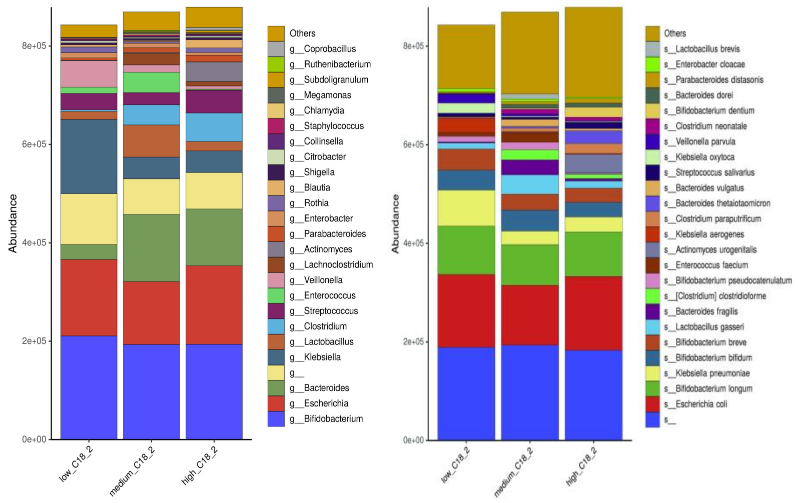

C18:1

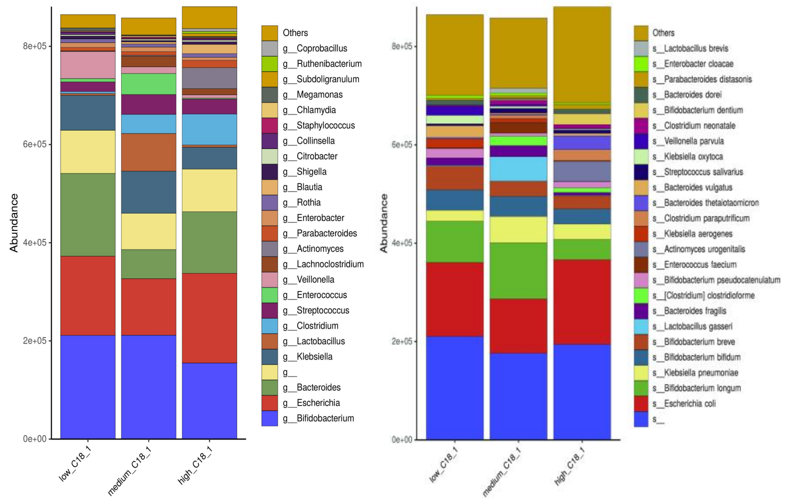

C18:0

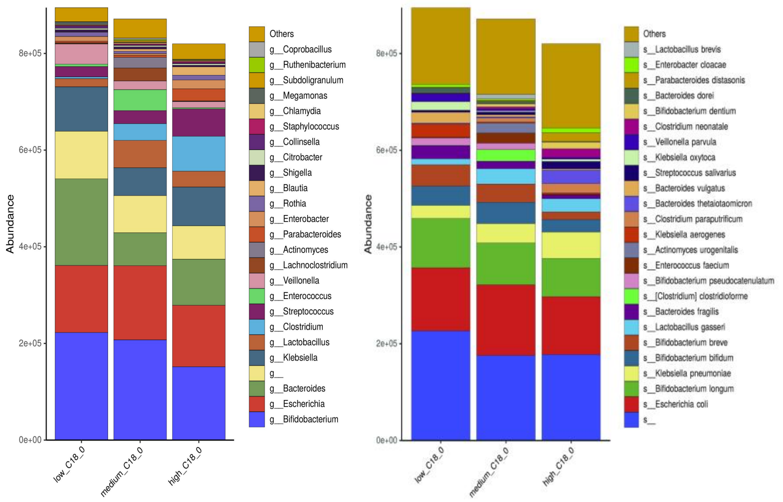

C16:1

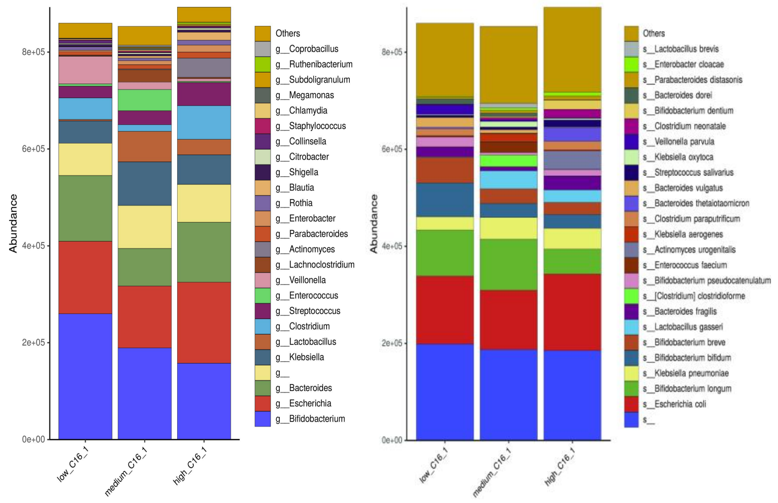

C16:0

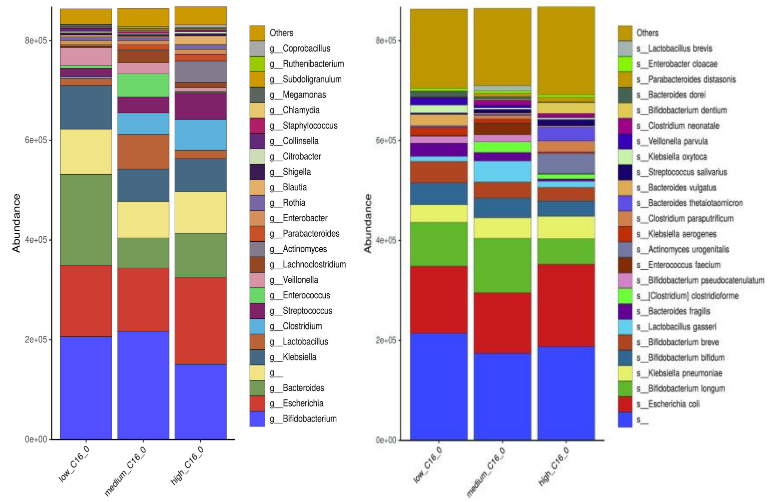

C15:0

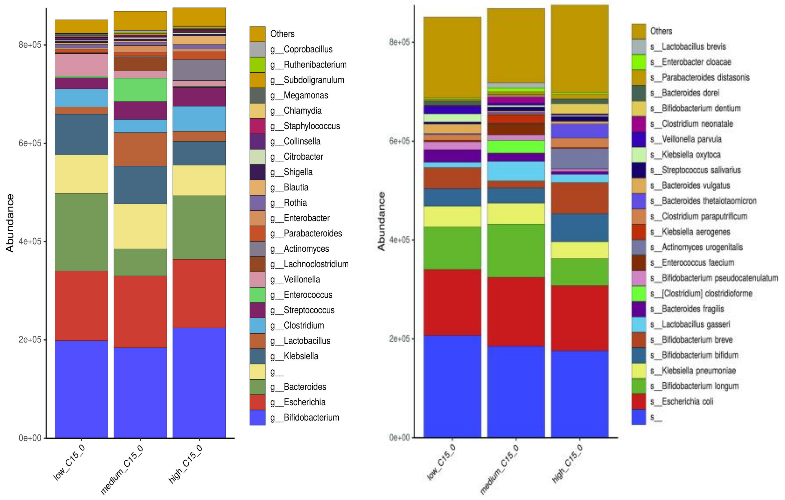

C14:1

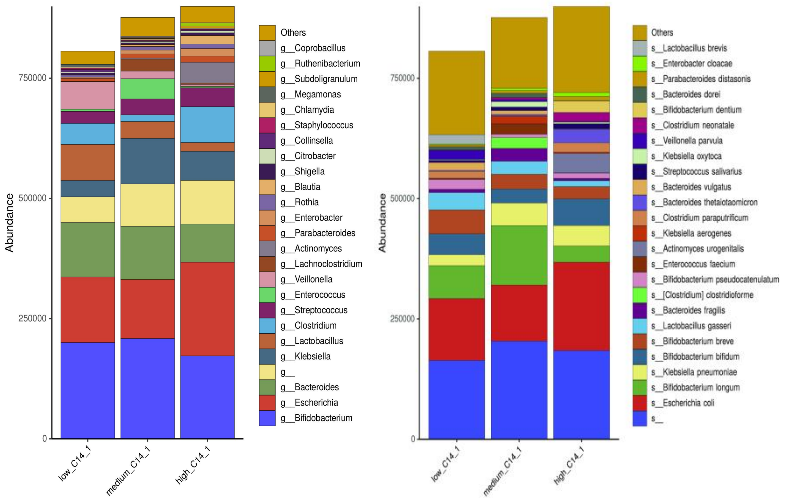

C14:0

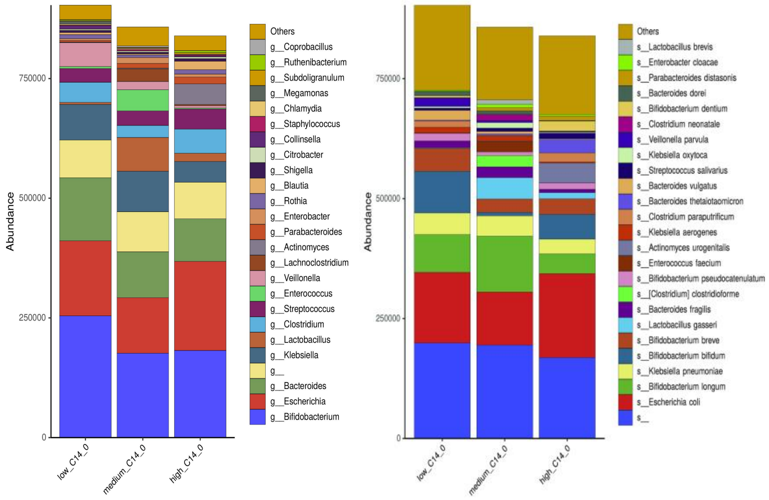

Figure S3. The relative contributions of the top 30 genera and the species present in each group.

Table S1 Correlation between breast milk long-chain fatty acid content and breast milk fat intake

	**Fat intake**		**Fat energy supply ratio**	
	correlation coefficient	*P*	correlation coefficient	*P*
**C22:6**	0.070	0.608	0.197	0.145
**C22:5**	0.004	0.974	0.186	0.169
**C22:4**	-0.008	0.955	0.212	0.116
**C20:5**	-0.030	0.827	0.123	0.366
**C20:4**	-0.009	0.950	0.205	0.129
**C20:3**	-0.044	0.748	0.121	0.376
**C18:3**	0.006	0.967	0.101	0.461
**C18:2**	0.020	0.882	0.084	0.539
**C18:1**	-0.032	0.817	0.104	0.446
**C18:0**	0.084	0.539	0.094	0.491
**C16:1**	-0.011	0.938	0.177	0.193
**C16:0**	0.018	0.893	0.110	0.420
**C15:0**	-0.038	0.779	-0.048	0.725
**C14:1**	-0.014	0.920	0.140	0.304
**C14:0**	-0.011	0.935	0.034	0.805
